# An appraisal of data collection, analysis, and reporting adopted for water quality assessment: A case of Nigeria water quality research

**DOI:** 10.1016/j.heliyon.2021.e07950

**Published:** 2021-09-06

**Authors:** Ugochukwu Ewuzie, Nnaemeka O. Aku, Stephen U. Nwankpa

**Affiliations:** a Analytical/Environmental Unit, Department of Pure and Industrial Chemistry, Abia State University, Nigeria; b Medical Microbiology Unit, Department of Microbiology, University of Nigeria, Nsukka, Nigeria; c Public Health Unit, Department of Community Medicine, University of Nigeria, Enugu Campus, Nigeria; d College of Pharmacy, Roseman University of Health Sciences, South Jordan UT, USA

**Keywords:** Descriptive statistics, Online instrumentation, Artificial intelligence, Inferential statistics, Data visualisation, Nigeria

## Abstract

The appropriate acquisition and processing of water quality data are crucial for water resource management. As such, published articles on water quality monitoring and assessment are meant to convey essential and reliable information to water quality experts, decision-makers, researchers, students, and the public. The implication is that such information must emanate from data obtained and analysed in an up-to-date, scientifically sound manner. Thus, inappropriate data analysis and reporting techniques could yield misleading results and mar the endeavours of achieving error-free conclusions. This study utilises the findings on water quality assessment in Nigeria over the last 20 years to reveal the likely trends in water quality research regarding data collection, data analysis, and reporting for physicochemical, bacteriological parameters, and trace organics. A total of 123 Web of Science and quartile ranked (Q1–Q4) published articles involving water quality assessment in Nigeria were analysed. Results indicated shortcomings in various aspects of data analysis and reporting. Consequently, we use simulated heatmaps and graphs to illustrate preferred ways of analysing, reporting, and visualising some regularly used descriptive and inferential statistics of water quality variables. Finally, we highlight alternative approaches to the customarily applied water quality assessment methods in Nigeria and emphasise other areas of deficiency that need attention for improved water quality research.

## 1 Introduction

Water quality studies are probably among the most important ventures in the field of environmental studies in the 21^st^ century. This perception may be connected to the fact that water for various human endeavours such as industrial use, agriculture, construction, consumption, or other domestic applications demands a certain degree of quality to assure the suitability for the anticipated use. Over the years in Nigeria, the parameters of interest in water quality monitoring and assessment have been in the groups of physicochemical (Afonne et al., 2020; Alum and Okoye, 2020; Bello et al., 2020; Beshiru et al., 2018; Egbueri, 2020; Ewuzie et al., 2020; Omaka et al., 2016), bacteriological (Aboh et al., 2015; Adesakin et al., 2020; Aromolaran et al., 2019; Bamigboye et al., 2020; Chigor et al., 2012), and trace organics, including other emerging contaminants of concern (Aganbi et al., 2019; Ebele et al., 2020; Ogbeide et al., 2019; Ogunbanwo et al., 2020; Sogbanmu et al., 2019).

The contents of these parameters determine the water quality because the index for categorising water as suitable or unsuitable for the intended use is usually based on their concentrations (Nnorom et al., 2019). Copious research studies have focussed on and provided evidence about the ubiquity of various potentially toxic elements (PTEs) in aquatic systems in Nigeria, and at times, their levels are above the permissible limits (Nganje et al., 2020; Omaka et al., 2017). Chemical contaminants such as PTEs pose health risks to water consumers, while physical parameters such as colour, taste, and odour make water unpleasant to drink (WHO, 2017). To illustrate, PTEs such as As, Cd, Hg, and Pb have no health benefits when ingested through drinking water; rather, they are associated with cardiovascular disorders, renal injuries, and cancer risk in humans (Yao et al., 2021). Also, they can induce neurotoxicity, oxidative stress, and alter immune systems in aquatic animals (Ihunwo et al., 2020).

Trace organics, including other recently identified groups of emerging contaminants of concern have been detected in various water bodies worldwide. For instance, pharmaceuticals and personal care products were detected in some water bodies in Egypt (Abou-Elwafa Abdallah et al., 2019), Spain (Sadutto and Picó, 2020), and China (Lin et al., 2020). Also, Ramírez-Morales et al. (2021) reported the levels of pesticides in surface water in Costa Rica; in India, emerging contaminants such as endosulphan and hexachlorohexane were detected in some groundwater samples (Vashisht et al., 2020). In Nigeria, studies are increasing on the trends and occurrence of these recalcitrant chemicals in the water bodies (Ebele et al., 2020; Ogbeide et al., 2019). Most of these chemicals have been implicated in endocrine disruption, thyroid function suppression, increased risk of developing diabetes, hormonal imbalance, and adverse neurobehavioral development (Carpenter, 2011; Ukaogo et al., 2020).

Evidently, the bacteriological assessment of domestic water– to determine water quality– is essential from a public health perspective. The results of such evaluations allow for informed actions to be taken by the relevant authorities, and ultimately, to prevent the outbreak of water-borne diseases such as cholera, diarrhoea, typhoid, and gastroenteritis (Adesakin et al., 2020; Ayandiran et al., 2018; Dahunsi et al., 2014; Ukaogo et al., 2020). Certainly, these contaminants deteriorate water quality, render water resources unsafe for human consumption, and create uninhabitable environment for aquatic animals (Kolawole et al., 2011). Therefore, studies assessing the levels of these contaminants must be stringent with their data collection and analysis, and communicate their findings in an accurate and compliant manner.

Incidentally, not all the studies in published articles can boast of adhering to the recommendations in the standard protocols regarding data processing for accurate communication of findings. Statisticians documented that statistical errors have become common in the scientific literature; so much so that, about 50% of published articles have at least one statistical error (Altman and Bland, 1991; Curran-Everett and Benos, 2004). This concern possibly stimulated some journals to publish editorials and offer guidelines for communicating research findings. An instance is the ‘Guidelines for reporting statistics in journals published by the American Physiological Society’ (Curran-Everett and Benos, 2004). One of the enumerations of the guidelines, ‘*Analyse your data using the appropriate statistical procedures and identify these procedures in your manuscript’* buttresses the importance of applying adequate procedures and communicating them in scientific articles. Invariably, the non-reporting of important procedures in not only statistical evaluations but also in data collection and reporting in environmental sciences is becoming more common. This situation is worrisome, in that, procedures not reported might be construed as procedures not followed. Therefore, it is high time experts took cognizance of this trend and nip it in the bud before it becomes a new normal.

In Nigeria, water quality monitoring and assessment have been well documented in the scientific literature. A recent review presented a two-decade literature analysis of water quality monitoring and assessment in Nigeria, focussing on pollution sources of rainwater, ground, and surface water sources (Ighalo and Adeniyi, 2020). This review synthesized the findings in the literature in a systematic manner and communicated that rainwater was less polluted than surface and groundwater in most parts of the country. Such deductions serve as veritable information not only to the government and water quality experts but also to the general public. Furthermore, people depend on such information to choose the type of water they consume, since it is rarely on the priority list of the Nigerian government to provide them with treated water (Nnorom et al., 2019). Therefore, it is pertinent that the data used for such conclusions are obtained and analysed in a scientifically sound way.

One of the concepts that has not received much attention in water quality assessment in Nigeria is the subject of this discourse, i.e., appraising how data generated in water quality assessment are analysed and reported. Several reviewed articles tend to follow the approach of previous articles when it comes to the mode of data acquisition, the choice of statistics, and the use of regular graphics such as multiple pie charts and stacked bar plots. At times, these graphics may be unwieldy and provide little ability to compare differences between data groups; apparently, there is a certain reluctance to change or improvement. A possible reason for this practice is the availability of standard statistical packages with pre-loaded analytical and graphical options. These seemingly simplified tools increase the unwillingness of most researchers that are not core statisticians or computer scientists to acquire some programming skills to create innovative graphics in environments such as MATLAB and R. However, an important theme, which this article seeks to promote, is the proper knowledge and appropriate use of statistical tools in water quality data analysis.

There are problems with incorrect statistical analysis of water quality data. For instance, variables can appear unrelated when there are fascinating relationships, or their real interrelationship may not be correctly inferred. Again, variables may seem to cluster, but really, they belong to different groups, thereby leading to misclassification. Furthermore, incorrectly reporting numerical data can result in misinterpretation. For example, reporting Pb concentration in drinking water as “ND” (not detected) without giving the corresponding quantification or method detection limit may be misleading. The aftermath is that an unsuspecting and/or amateur reader may interpret “ND” as not present in the water since it was not detected. Besides, using standard deviation to quantify the dispersion of skewed data is of questionable value for water resources data (Helsel et al., 2020).

Water quality research entails collecting data through online (remote) instrumentation, in situ measurements, and/or laboratory experimentation, then analysing the data using appropriate statistical techniques, and subsequently interpreting and reporting the results. Data collection is the basic and most crucial step in water quality research. It has the objective of ensuring that dependable and informative data are collected for statistical analysis so that, in the end, useful decisions for the protection of water quality can be reached. The entire data collection process borders on how methods are selected, implemented, and quality-assured for water quality-related field and laboratory measurements (USEPA, 2000). Various consensus-based standards organizations (USEPA, 1999; USGS, 2010; WHO, 2017) and other literature (Matamoros, 2012; Tovar-Sánchez, 2012) have provided excellent guidance on methods for data collection for water quality assessment. However, a more recent publication of the United States Geological Survey (Helsel et al., 2020) provides a guide for statistical analysis in this field.

This article, therefore, is not intended to be an exhaustive treatise on data analysis and statistical concepts, and we suggest that readers consult various articles and statistics books for a thorough understanding of these concepts. Thus, this study attempts to overview the statistical analysis of data generated in water quality assessment studies in Nigeria and the reporting styles. In addition, specific examples as well as visuals and heatmaps were simulated to showcase the preferred applications of descriptive and some inferential statistics to establish uniform and acceptable data analysis and reporting patterns, which will be applicable not only for water quality assessment but also to environmental studies in Nigeria and other countries. To accomplish this, we reviewed articles on water quality assessment published in the last 20 years in Nigeria. By doing this, we intended to integrate the current knowledge and identify common gaps and drifts in data analysis and reporting patterns. Finally, alternative approaches to the customarily applied water quality assessment methods in Nigeria are highlighted, and other areas of deficiency that need attention for improved water quality research are emphasised. Of relevance to this review is attention to appropriate data analysis, which will open new fascinating research directions and revamp the confidence of water quality experts, researchers, students, decision-makers, and the public on water quality research in Nigeria and other countries.

## 2 Methods

### 2.1 Nigeria's water resources

Nigeria is adjudged a nation with the most populated black humans in the world (Ighalo and Adeniyi, 2020); and according to the latest data (the year 2020) from United Nations Population Fund (UNFPA), has a population of about 206 million people, making it the 7^th^ most populated nation in the world (https://www.unfpa.org/data/world-population/NG). Nigeria's hydrological (occurrence and distribution of ground and surface water resources), meteorological (seasonal variation in temperature, rainfall, humidity, and general atmospheric weather condition as well as how they influence water quality), and geological (diverse lithostratigraphic units found in different regions and their rock-water interactions) blueprints have been well documented in recent literature (Ewuzie et al., 2020; Ighalo and Adeniyi, 2020; Nnorom et al., 2019).

In this context, Nigeria's water resources are further examined to divulge the possible rationale behind the disparity in the number of studies related to water quality in different regions of the country. Already, the amount of annual rainfall disproportionately favours the southern regions of the country against their northern counterpart, which is a result of the nearness of the former to the Atlantic Ocean, and the latter to the Sahara Desert (Ighalo and Adeniyi, 2020). Consequently, the south-south and south-west have abundant surface and groundwater, and annual rainfall is high. Specifically, the major sources of water in the south-south are creeks, rivers, shallow wells and boreholes; and studies of water quality in this part have been tailored to them (Ihunwo et al., 2020; Ocheli et al., 2020; Owamah, 2020; Owoyemi et al., 2019). Similarly, studies in the south-west have been related to several rivers, lagoons, groundwaters, lake and stream, as these are abundant (Ebele et al., 2020; Ezemonye et al., 2019; Gbadebo, 2020; Odukoya et al., 2017; Ogunbanwo et al., 2020). In the south-east region, groundwater in the form of dug-wells, boreholes and springs, as well as streams, lakes, and few rivers have been studied (Alum and Okoye, 2020; Chukwuka et al., 2019; Egbueri, 2020; Ekere et al., 2019; Ewuzie et al., 2020; Nganje et al., 2020). On the contrary, due to the scarcity of water in the northern region, wells, dams, few rivers and groundwater are the major sources of water and have been studied (Jagaba et al., 2020; Okunola et al., 2020). A case in point is that besides other factors such as researcher's area of residence, availability of funds for research, accessibility of functional laboratories with requisite equipment, and research interest and field of study; the abundance of different water sources to be investigated in some regions could be linked to the copious research projects and publications from such regions. Moreover, this is corroborated by the findings of Ighalo et al. (2020) who added that literacy level and quality of higher institutions of learning are among the reasons why research output in water quality assessment varied by geopolitical zones in Nigeria.

### 2.2 Dataset collection

Three well-known search engines, Google Scholar (https://scholar.google.com/), ScienceDirect (https://www.sciencedirect.com), and PubMed (https://pubmed.ncbi.nlm.nih.gov) were employed to systematically comb for published articles on water quality assessment in Nigeria. All these search engines possess a succinct search “timeline” that enables the search of documents within the desired range of years, and at the same time, informs how many papers, relative to the search criteria, are contained in each year. The keywords used were “Quality”, “Water” and “Nigeria”, searched simultaneously as “water quality in Nigeria”. The use of these keywords ensured that papers germane to the review were obtained as revealed by other researchers (Ighalo and Adeniyi, 2020). The search was completed between 24^th^ and 26^th^ October 2020. During the search, the papers were filtered to comprise the date range of 2000–2020. We downloaded a total of 183 research papers (excluding reviews and book chapters) published within the time frame and dealing with water quality assessment of surface, groundwater, rainwater, and sachet/bottled water in Nigeria. However, the number of the papers was reduced to 123 after papers that were not published in Web of Science journals and not quartile-ranked (Q1–Q4) were dropped. This criterion was used to ensure the authenticity and traceability of the data presented and analysed in this review. Other details about the journals can be found in Table S1 (Supplementary Table).

### 2.3 Description of the collected dataset

**Figure 1:**
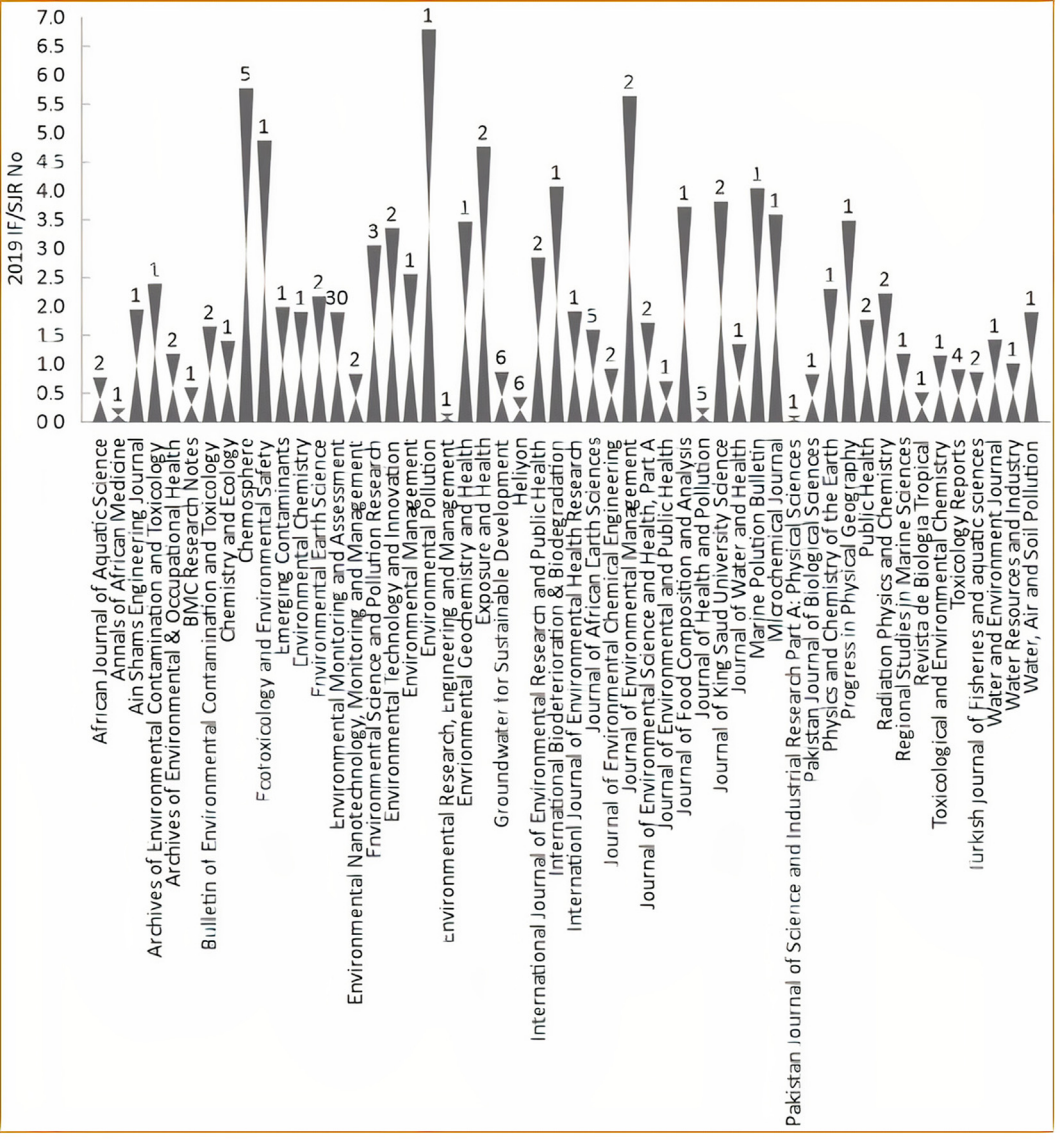
List of the journals showing the number of the reviewed articles in each journal and 2019 impact factor (IF) or scientific journal ranking (SJR) of the journals.

One hundred and twenty-three research papers on water quality assessment, published in the last two decades in Nigeria made up the dataset for this review. Figure 1 depicts the journals in which authors have published water quality-related papers in Nigeria in the last 20 years, their impact factors, and the number of reviewed articles in each journal. Figure 1 reveals the quality and strength of the journals, which communicated the research findings of water quality assessment in Nigeria. It is also indicative of the journals that were frequently patronized in the last 20 years. For instance, approximately 24.4% of the reviewed papers (30 out of 123 articles) were published in *Environmental Monitoring and Assessment* journal, whereas other higher impact factor journals such as *Environmental Pollution, Chemosphere, Journal of Environmental Management, and Ecotoxicological and Environmental Safety* had equally received papers on water quality assessment in Nigeria in the last 20 years.

**Figure 2:**
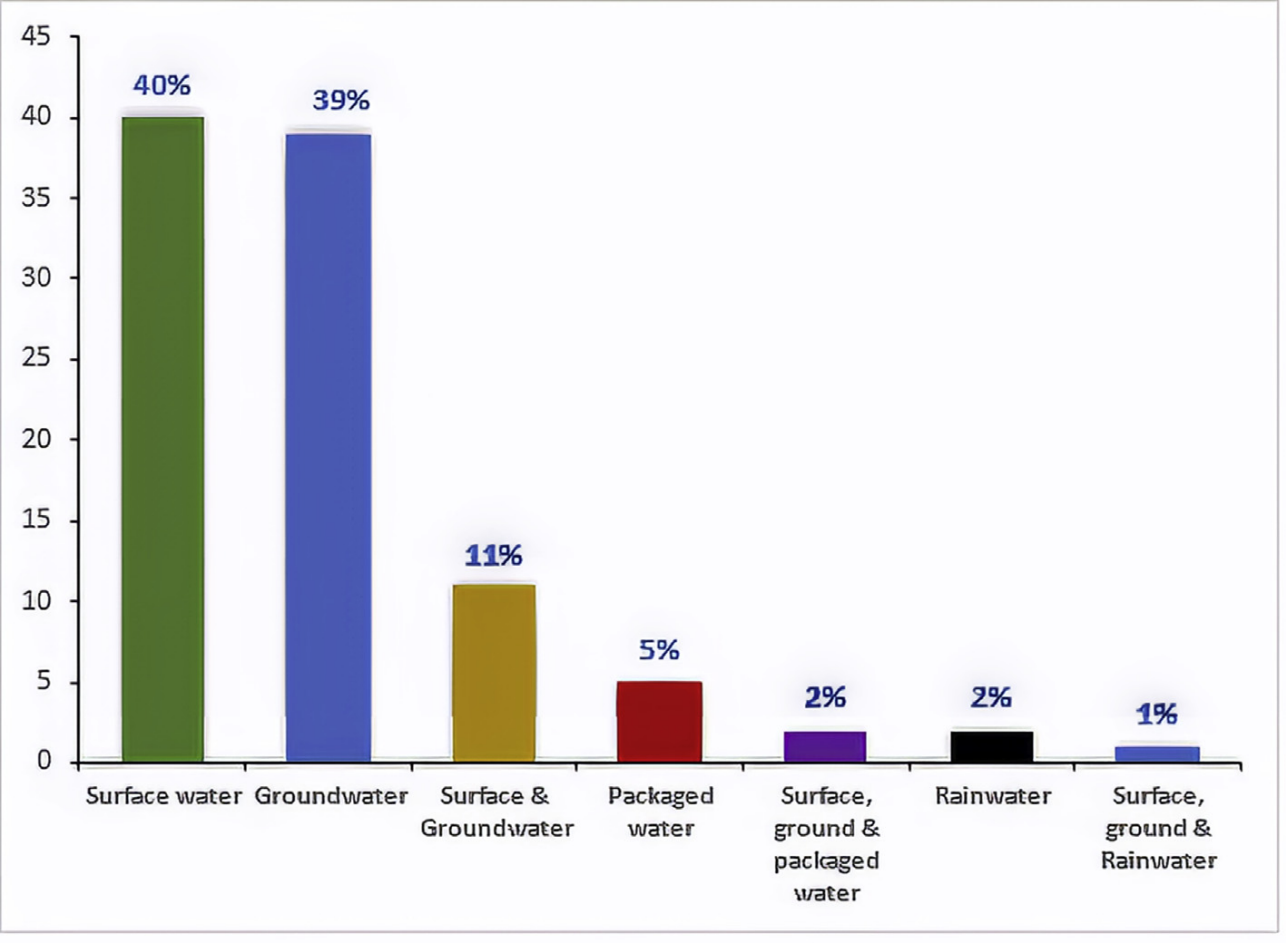
Percentage allotment of the reviewed papers with water sources in perspective.

More importantly, the water sources considered in the reviewed papers, which are the sources of water in Nigeria are surface (river, pond, stream, lagoon, lake, dam, creek, reservoir), groundwater (borehole, well, spring), packaged water (in sachets, bottles), and rainwater. Among these sources, rainwater had a dearth of papers on its quality as depicted in Figure 2. This could be because of fewer people who use this source of water. Besides, the usage is often limited to the rural areas where surface water may be in abundance, thus, making rainwater an unpopular source. Packaged water in Nigeria comes in sachets or bottles and is marketed in both rural and urban cities of the country, though the latter takes the larger proportion. Considering the proliferation of the use of this source, the literature on its quality in the last two decades is scanty, as shown in Figure 2. Nevertheless, this article does not intend to compare the water sources but rather appraise the data analysis and reporting approaches employed in the studies of all the water sources.

**Figure 3:**
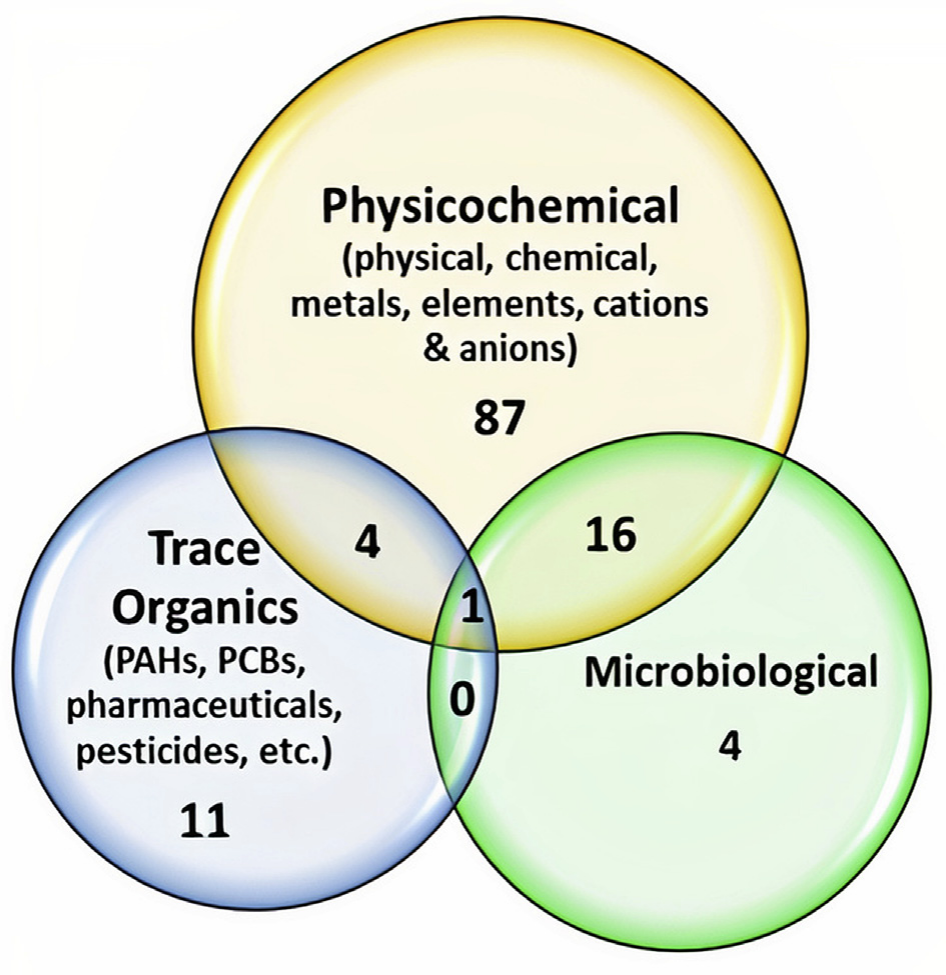
Venn diagram showing the number of reviewed papers studying different parameter combinations.

Overall, the selected papers contained data on physicochemical parameters (including physical, chemical, elements, metals, anions, and cations), some trace organic contaminants of emerging concern (including pharmaceuticals, pesticides, polychlorinated biphenyls, polycyclic aromatic hydrocarbons, phenol, formaldehyde, and others) and bacteriological indicators. By chemical, we mean other parameters such as ammonia, hardness, alkalinity, chemical oxygen demand (COD), and biochemical oxygen demand that are different from metals or elements. Also, the trace organic classes studied in the reviewed articles are included in Table S2. The number of the reviewed papers in the last two decades and the allotment of the parameters are elucidated in a Venn diagram (Figure 3). This figure shows that these studies, over the years, have focussed more on the physicochemical contents of these water sources. Thus, this contaminant class was mainly used to illustrate preferred applications of statistical analysis and reporting of water quality data, while their applications for the other contaminant classes (i.e., bacteriological and trace organics) were evaluated.

## 3 Review results and discussion

This section presents the results and discusses the findings from the reviewed papers. It is divided into three sub-sections corresponding to the items in the theme of this review, namely, data collection, data analysis (statistical approaches), and reporting and visualisation.

### 3.1 Data collection

Collection of water quality data, either in the field or through laboratory experimentation usually precedes data analysis and reporting of results. In situ real-time (online) data acquisition is highly desirable in that it provides early detections of deviations or problems, facilitate proactive management of water supply systems while reducing the labour and cost associated with laboratory measurements (Bridgeman et al., 2015; Forzani et al., 2007). On the other hand, data collection through laboratory experimentation is required for regulatory purposes (Bridgeman et al., 2015), and in most cases where online instrumentation for water quality monitoring is non-existent. Besides, data collected by the online instruments are usually compared/verified with those from laboratory experimentation, at least at the developmental stage (Szabo and Hall, 2014). Most of these monitoring tools are based on sensor technology and can be used to collect data in situ (online) or after sample collection (Belikova et al., 2019).

Much earlier, most fabricated sensors were mainly for the collecting water quality data of physical nature such as temperature (Culler et al., 2004). But recent advances in optical fibre and nanomaterial-enabled screen-printed electrochemical sensors have enabled the collection of chemical or biological data such as trace metals (Liu et al., 2019; Mimendia et al., 2010; Pesavento et al., 2019) pesticides (Saleh et al., 2020), and microbial and organic matter (Bridgeman et al., 2015). Additionally, a water quality automatic monitoring system based on general packet radio service (GPRS) data communications can continuously acquire real-time data and send them to control centres or personal gadgets (Ionel et al., 2015). These wireless instrumentations have also been successfully implemented in collecting water quality data (e.g., temperature) for the oil and gas industry (Petersen et al., 2014), and are advantageous in environments where data collection points are scattered (Wei et al., 2012). Besides, the clamour for data collection through online equipment is due to their swift methods to aid the development of operational response and provide a level of public health protection in real-time (Storey et al., 2011). Also their methods are fully automatic and considerably minimize the use of reagents, thereby contributing to the so-called green chemistry (Rodríguez-Maese et al., 2020). Conversely, some authors have expressed concerns that using these technological devices may enable the collection of much more data than required. This notion is due to the ease with which data can be collected, for example, in a water quality assessment or monitoring program, thereby wasting time that would have been useful for data analysis and reporting (Falkenberg and Styan, 2014; Hanley, 2012).

More importantly, all the reviewed articles implemented data collection either through laboratory experimentation or portable monitoring devices, but not remote online instrumentation. Irrespective of the data collection method, it should follow the guidelines in standard protocols (such as USEPA, 1999; USGS, 2010; WHO, 2017) for data collection to ensure that the data collected are dependable and adequate, which when analysed, would be informative for decision-making. Nevertheless, the evaluation of whether the reviewed articles implemented these guidelines or not is elaborate and not within the scope of this review. Still, we assumed adequate data collection for the articles that documented the standard protocol or literature they adopted as a guide for collecting their data. Based on this conjecture, we identified some international (APHA, USEPA, ASTM, AOAC) and national (Nigeria Department of Petroleum Resources, DPR) protocols as well as literature that served as references for data collection. For the articles on physicochemical parameters, approximately 58.3% cited at least one of the international or national protocols; about 14.8% employed the procedures found in the literature, while the remaining 26.9% did not report adopting the guidelines in any standard document for data collection (Supplementary Table S3). In other words, approximately 73.1% of the reviewed articles supposedly used a reliable data collection approach, while the remaining articles might have acquired their water quality data in an undependable manner.

Also, for trace organic parameters, about 68.8% of the reviewed articles followed the procedure prescribed by standard organizations (such as USEPA, APHA) or the literature for their data collection. Nonetheless, the other 31.2% did not present such a report. Again, reviewing the articles that studied bacteriological parameters, it was observed that 55% of them collected their data in a manner that conformed with guidelines in the standard protocols. Conversely, almost half of the articles (45%) possibly used a less reliable approach during data collection. In scientific writing, it is essential to detail the methods and protocols employed during data collection and analysis; otherwise, it may indicate that no standard protocol was used as guidance, thereby discrediting the work.

### 3.2 Statistical data analysis

Hardly had a water quality assessment study been adjudged complete when the statistical analysis of the generated data had not been done. This process positions the concept of data analysis at a higher priority in water quality research. This sub-section takes a critical look at how the reviewed articles analysed the data generated in their studies. It is discussed under the following headings: descriptive statistics, classification of variables, inferential statistics, and data pre-treatment.

#### 3.2.1 Descriptive statistics

Descriptive statistics aims to allow for data presentation in a meaningful and understandable way, and ease of interpretation. Appropriate knowledge of descriptive statistics is key in presenting/summarizing a set of observations as simply as possible, which at first glance, communicates the salient details of the dataset, such as the symmetry of the distribution. The United States Geological Survey document (Helsel et al., 2020) described water resources data as being characterised by the presence of censored values (otherwise referred to here as rounded zeros), non-normal distribution of data, presence of outliers, and skewness of data; the last three being interwoven. Mean (also known as the arithmetic mean; average) and median are the most popular measures of central tendency used in water quality assessment, while standard deviation (SD) and standard error (SE; also called standard error of the mean) are the most used measures of dispersion for water quality data, at least in Nigeria. From the reviewed articles, it appears that researchers almost always compute mean and SD, and rarely the median, as descriptive statistics (See Table S3). Krzywinski and Altman (2013) had observed such practice and stated that researchers use the SD or SE with approximately equal frequency in research papers. However, Greenacre (2016) pointed that *“this way of thinking about the SD as a single all-purpose measure of the spread of the data is clearly inappropriate in the case where the data values are highly skewed”*.

The reason why the mean as a measure of central tendency may not be preferred in presenting skewed data is that all observations do not have the same influence on the mean; thus, the mean may not give an intuitive summary of the “centre” of the data. Therefore, computing a resistant estimator of central tendency such as the median with other quantiles when dealing with skewed data may be preferable, since the median is only minimally influenced by the magnitude of any single observation (Helsel et al., 2020; Manikandan, 2011). It was observed from the reviewed articles that only a few articles used the median due to the skewness of their data for physicochemical parameters (Aremu et al., 2002; Ebele et al., 2020) and trace organics (Ebele et al., 2020). Again, a few other articles computed both the mean and median (Edet and Worden, 2009; Nganje et al., 2015; Nnorom et al., 2019), which illustrates a better usage of the statistics for skewed data. Similarly, SE and SD describe the variability of the mean of a group of data and the dispersion of the original data, respectively. However, just like the mean, the SD can be beneficial for the said purpose only when data are distributed symmetrically (and especially normally distributed) about the mean. This postulate is so because the values are calculated using the squares of deviations of data from the sample mean; thus, extreme values affect their magnitudes. The consequence of using SD at such an instance is that it may give the impression of higher variability than most of the dataset indicated. Nevertheless, authors have reported that they compute the arithmetic mean to compare their results with guidelines (usually mean values) for individual parameters (Nnorom et al., 2019). While this may be true, in our own opinion, it should be indicated in the manuscript, and the appropriate measure of central tendency be computed and included in the results as well. Furthermore, other proper measures of dispersion (other than SD or SE) that are not influenced by outlying observations include the use of quantiles (interquartile range, percentiles) and median absolute deviation (MAD) (Ewuzie et al., 2021; Helsel et al., 2020). The margin of error (ME) is more useful than the SE, since it gives the bounds of a confidence interval for the mean (Greenacre, 2016).

#### 3.2.2 Classification of variables

Classification of variables through cluster analysis helps to agglomerate several variables or observations into a number of meaningful homogeneous groups (Ewuzie et al., 2021). Cluster analysis (CA) is commonly used in water quality assessment studies, and approximately 11.1% of the reviewed articles on physicochemical parameters performed this analysis (Table S3). In most cases, the variables that are being grouped have different units or orders of magnitude. Thus, using the standard statistical applications for such data during CA, for example, without transformation, may result in misclassification. Data transformation (via *z*-score standardisation or range scaling (normalisation)) is usually applied before CA. Nevertheless, statistical computing in an environment such as R can simultaneously perform the required standardisation and cluster analysis. Standardisation or normalisation of data serves the purpose of jettisoning the unit limit of data and transforming them into a totally dimensionless value, after which various units or orders of magnitude can be weighted or compared (Li et al., 2019). It is very useful for cluster analysis where wide differences in data dimensionality could cause misclassification (Liu et al., 2003; Omo-Irabor et al., 2008). The z-score standardisation is performed by subtracting the mean value from each variable score and dividing by the standard deviation (Equation 1). In such a case, each standardised value would reflect the distance from the mean in units of standard deviation.

(1) $$\text{z}-\text{score}\;\text{standardised}\;\text{value}=\frac{\text{Variable}\;\text{score}-\text{mean}}{\text{Standard}\;\text{deviation}}$$

From our discussion in section 3.2.1 about the use of mean and SD, Eq. (1) shows that unless the data are normally distributed, z-score standardisation before cluster analysis may still result in misclassification (This will be graphically elucidated in section 3.3). However, if it is assumed that all variables have come from approximately normal distribution, then z-score standardisation would bring them all close to the standard normal distribution. Thus, the resulting distribution will have a mean of 0 and a standard deviation of 1. Nevertheless, suppose it is pre-determined that the variables have possibly emanated from different (and non-normal) distributions. In that case, range scaling (normalization) may be applied to brings the data to the 0 to 1 scale, which is done by subtracting the minimum value from each variable score and dividing by the range (Equation 2).

(2) $$\text{Range}\;\text{scaled}\;\text{value}=\frac{\text{Variable}\;\text{score}-\text{Minimun}}{\text{Maximun}-\text{Minimum}}$$

For the reviewed papers on physicochemical parameters, only 4 out of the 12 papers that performed cluster analysis standardised their data before the test, while the remaining articles did not mention transforming or standardizing their multivariate data that included variables on different scales/units. Furthermore, a few articles on trace organics performed cluster analysis but there was no report on transformation of their data that might have been skewed (Adekunle et al., 2017; Omo-Irabor et al., 2008). More so, in the reviewed articles as well as other studies conducted in other countries (Table S4), z-score standardisation seems to be the only method of transformation before CA and principal component analysis (PCA), even when it was determined that the data distribution was non-normal. This approach may not be appropriate, especially for highly skewed data that might likely influence z-score value whose determination is dependent on the mean and SD (Equation 1). More importantly, adequate knowledge of the generated data is necessary to perform pre-treatment tests that are only relevant to the statistical analysis to be done. For instance, over 85% of the reviewed articles might have not standardised or normalised their data because they did not perform any analysis (such as CA) that warranted such a procedure or that their data are in a uniform scale with negligible variances.

#### 3.2.3 Inferential statistics

As the name implies, they are used to reach conclusions pertaining to the population by observing the sample data. In most cases, they offer a quantitative method regarding the acceptance or rejection of the null hypothesis. Numerous statistical tests are available for this purpose; the test chosen should be dependent on the type of data being analysed and the number of groups involved. In water quality assessment, we are sometimes concerned with contaminant effects and whether a particular contaminant is as impactful as another in a water source. Likewise, the interest may lie in knowing whether the contaminant's average level varies in significant amounts in different water sources. In other words, a dependent variable in a particular case may be an independent variable in another scenario. Such studies and many others designed to answer these questions depend on inferential statistics to disprove or support one treatment over another. Furthermore, assessing whether the association of parameters is significant or not also relies on inferential statistics. In the reviewed articles, sample t-test, correlation analysis, and analysis of variance are some of the significant inferential statistics employed, sometimes in error.

##### 3.2.3.1 Correlation and comparison of group means

Studying interrelationships through correlation and comparing group means through t-tests or analysis of variance (ANOVA) are among the widely computed statistics in water studies in Nigeria. Linear correlation is used to evaluate the association between two variables, which is reflected by the value of the correlation coefficient (Misra et al., 2021). In water quality research, a high positive correlation (r > 5) between two variables such as Pb and Hg suggests that both elements can have similar hydrochemical characteristics and vice versa (Nnorom et al., 2019). Furthermore, a t-test is applied for comparing the means of two groups of data; it determines whether the means of the two groups are equal (Helsel et al., 2020). ANOVA is analogous to a t-test between three or more data groups and is governed by the same assumptions as to the t-test (Williams and Quave, 2019). When two groups of elements such as Fe and Mn have equal means, it may suggest that they have similar origin (Nnorom et al., 2019). In performing any of these parametric tests, it is expected that the distribution of the data or residuals will at least be approximately normal. However, their corresponding non-parametric tests are available for use when non-normality is suspected/ascertained. For instance, Spearman correlation instead of Pearson correlation, Wilcoxon Rank test in place of two-sample t-test, or the Kruskal Wallis test instead of ANOVA should be used (Williams and Quave, 2019). These nonparametric tests have been said to exhibit greater power than do parametric tests in the presence of skewness and outliers (Helsel et al., 2020). Another alternative is the transformation of skewed data, after which parametric tests can be used (data transformation is discussed in section 3.2.4). The bottom line is that it is difficult to say how sample data or residuals are distributed without either visualising or testing them. Generally, visual (stem-and-leaf plot, boxplot, P–P plot, Q-Q plot) and statistical (Anderson-Darling test, Cramer-von Mises test, Anscombe-Glynn kurtosis test, D'Agostino skewness test, Kolmogorov-Smirnov (K–S), Lilliefors corrected K–S, and Shapiro-Wilks test) methods of testing for normality are well documented (Öztuna et al., 2006). Among these tests, the Shapiro-Wilks test, which is based on the correlation between the data and the corresponding normal scores, offers more acceptable power than the K–S test even after applying Lilliefors correction (Ghasemi and Zahediasl, 2012). Although their suitability depends on sample sizes and the properties of the data.

Whether to apply a preliminary test for normality or not is a question many researchers ask, but the point is knowing when to carry out this test for a particular statistical task. For example, while one of the assumptions of a correlation test is that the sample data should approximate a normal distribution, the assumption for testing between groups means (using t-test or ANOVA) is that the residuals should be approximately normal. In light of the foregoing, it means that preliminary testing of data for normality is performed only before a correlation test; ANOVA or t-test is performed first before adequately using any of the graphical or statistical methods to ascertain the normality of the residuals. This is another aspect that was greatly misconceived in almost all the reviewed articles. All the articles that performed the test for normality did so before any parametric test. They reported testing for normality, which enabled them to choose between parametric and nonparametric tests. While this is appropriate for a correlation test, it may be an unreliable approach for ANOVA and t-tests, in which the residuals need to be normally distributed too. Besides, some articles even reported using parametric tests after confirming the non-normal distribution of their data, probably due to their large sample sizes. Sample size has been recognized as an important factor in water quality studies since the data are usually non-normally distributed (asymmetric distribution). It is documented that as sample sizes increase (e.g., sample size ≥70), the distribution of the sample mean will be approximately normal (USEPA, 2002). However, sample sizes >30 have also been regarded as large enough for the assumption of the normality of mean (Ghasemi and Zahediasl, 2012; Greenacre, 2016). From the foregoing, it appears that some of the reviewed articles that performed one or more parametric tests without testing for normality did so because of their large sample sizes. The samples were large enough to allow the distribution of the sample means to be closely approximated by a normal distribution–Central Limit Theorem. For instance, twenty reviewed articles on physicochemical parameters that had sample sizes between 36 and 256 performed either ANOVA, Pearson correlation, or t-test without any report on normality test (Table S3). Conversely, 16 articles that performed one or more of these tests without testing for normality had sample sizes between 6 and 29. Thus, they might not have been lucky to have their sample mean approximating a normal distribution. We, therefore, recommend an appropriate normality check for small-sized data with obvious skewness, before carrying out a parametric test on the dataset.

#### 3.2.4 Data pretreatment

Water quality data are unique data. They are usually made up of variables with varying magnitude, non-detects (rounded zeros), outlying values, and even variables that can have negative integers such as redox potential. All these items make data analysis for water quality studies somewhat cumbersome. Data pretreatment is, therefore, an essential step in data analysis. It usually involves data imputation or replacement for missing values or rounded zeros, respectively, and data transformation for highly skewed data to achieve an appropriate scale of data before using standard statistical approaches. More importantly, the occurrence of samples with censored values for some parameters impacts the multivariate statistics for mapping hydrogeochemical processes (Carranza, 2011). Moreover, just imputing ‘zeros’ or values of the detection limits in statistical tools, especially when rounded zeros occur in large numbers may result in false correlation or even misclassification of clusters (Liu et al., 2003). Therefore, several ‘rounded zero’ replacement approaches have been proposed, including replacement with a fraction of the corresponding detection limit, for instance, 2/3 of detection limit (Carranza, 2011), statistical/mathematical models (Helsel, 2012), and even in developed software (Palarea-Albaladejo et al., 2014). Nevertheless, utilizing an inaccurate method may introduce bias; thus, researchers must select the appropriate method suitable for their censored data type.

Meanwhile, log-transformation and root transformation are the most used approaches to transform skewed water quality data. However, some issues regarding using the transformations mentioned above for skewed data have been raised (Ali and Bhaskar, 2016; Feng et al., 2014; Greenacre, 2016). These issues are related to the fact that log-transformation can only be used on positive data, arbitrary in the choice of root transformation, and dissimilarity in the back-transformed data and the original data, making it somewhat difficult for interpretation. Another drawback is the difficulty in finding a single transformation that can be applied to all groups, which will result in each having an appropriate scale with constant variance (Helsel et al., 2020). Regardless, log-transformation is widely used in water research, and when cautiously used, could produce data of appropriate scale, depending on the properties of the data. For instance, after log-transformation of initially asymmetrical data, it was confirmed that the skewness and kurtosis tests ranged from 0 to 0.060 (Mustapha and Aris, 2012), which indicated an approximately normal distribution of the dataset. More so, if a parametric test must be used to analyse small-sized and skewed data, then transformation is vital to achieving an appropriate scale. That way, the distributional assumptions of the parametric test will not be violated. Alternatively, use the modern resampling-based distribution-free bootstrapping method.

### 3.3 Data reporting and visualisation

Several published articles are already indicating (with evidence) the flaws in the reporting and presentation of results in fields such as ecology (Greenacre, 2016), rheumatology (Misra et al., 2021; Misra and Agarwal, 2020) physiology (Weissgerber et al., 2015), and statistics in general (Krzywinski & Altman, 2013, 2014); presenting appropriate alternatives. Studies on water quality should leverage these correct reporting recommendations to ensure that the efforts put in data collection and analysis are rewarded with informative interpretations. Therefore, this sub-section considers the reporting of descriptive statistics, both numerically and with graphical plots, visualisation of preferred approaches to CA and inferential analysis, and reporting of rounded zeros. To accomplish this, part of the dataset in one of the reviewed articles (Nnorom et al., 2019), downloaded from a repository in a public domain was used for simulation. The data can be found through the link: https://doi.org/10.17632/6dzmn2kb7m.1. Five trace metals (As, Co, Cr, Cu, Ni) from 24 stream samples (STR1–STR24) whose concentrations were in μg/L were chosen from the entire dataset. Bar plots with error bars (as SD) and boxplots were constructed in Statistica version 10. Also, heatmaps of preferred approaches to CA and some inferential analysis were simulated in R (R Core Team, 2020; https://www.R-project.org). The R scripts used for the illustrations are available to enable the readers to either reproduce or create their graphics in R.

#### 3.3.1 Reporting and visualisation of descriptive statistics

Apart from few reviewed articles that reported individual values of their results for physicochemical parameters (Omaka et al., 2017; Onwuka et al., 2019; Orisakwe et al., 2006; Ukah et al., 2018; Zacchaeus et al., 2020), and trace organics (Aganbi et al., 2019; Odukoya et al., 2010; Onyekwelu and Aghamelu, 2019), the others reported various measures of central tendency and dispersion (Table S3). The arithmetic mean was mainly reported irrespective of the nature of the data distribution, which is not a preferable approach (see comments on section 3.2.1). It is common knowledge that when data are skewed (which is common to most water quality data), the arithmetic mean is no longer considered equal to the median. Instead, the mean is on the side of the median where the tail of the distribution is longer. Therefore, the mean is typically larger than the median in any positively skewed data, and the standard deviation is likewise inflated by the long tail. Consequently, the tables of summary statistics comprising only the mean and standard deviation (or variance) are of questionable value for water resources data (Helsel et al., 2020). Thus, reporting the median and other percentiles such as interquartile range and MAD in summary tables, have much more applicability to skewed data.

Another misconception that has been dealt with in the literature (Greenacre, 2016; Krzywinski and Altman, 2013), which continues to manifest even in the reviewed papers is about the reporting of mean with plus/minus one standard deviation or one standard error (i.e., mean ± SD or mean ± SE). Readers should check the appropriate usage of SD and SE in section 3.2.1. One SD or SE does not give useful intervals because ±SD or ±SE only includes about 68% of the observations or 68% of the estimated dispersion of the mean, respectively. In the case of the dispersion of the mean, the ME, which is a critical value for the appropriate t-distribution multiplied by SE, gives about 95% confidence level, which is intuitive and signifies a high level of confidence about the mean's true value. Suppose the SD of the observed values must be reported for whatever reason. In that case, it should be written without the plus/minus prefix, for example, in brackets [i.e., mean (SD)] or separately. In all the reviewed articles, almost an equal number of articles reported mean ± SD or SE and the mean separately from SD or SE (Table S3). It is hoped that the clarification given above would help to address the inappropriate use of these statistics in water quality studies.

Graphical representation enhances the understanding of sample data and aid in making comparisons across samples. Nonetheless, inadequate presentation of data can either disguise or obscure vital information regarding the data. Visualisation such as stacked bar chart, bar plots with error bars, and multiple pie chart (without proper labelling) has been described as inappropriate owing to their inability to make a precise and easy judgement of differences as well as offer minute visual differences of groups of data (Helsel et al., 2020; Krzywinski & Altman, 2013, 2014). In our review, we found some articles that visually represented the variability of their data using bar plots with error bars (as SD) (Ejike et al., 2017; Emenike et al., 2020; Kolawole et al., 2011; Owamah, 2020) and dispersion of the mean using bar plots with error bars (as SE) (Ewuzie et al., 2020). However, the usage of error bars is strongly discouraged since they only reflect the variation of data (or mean) but not the error in measurement (Krzywinski and Altman, 2013). The preferred alternatives are the boxplot, quantiles, and the distribution-free bootstrap (see Davison and Hinkley, 1997 for bootstrap method).

**Figure 4:**
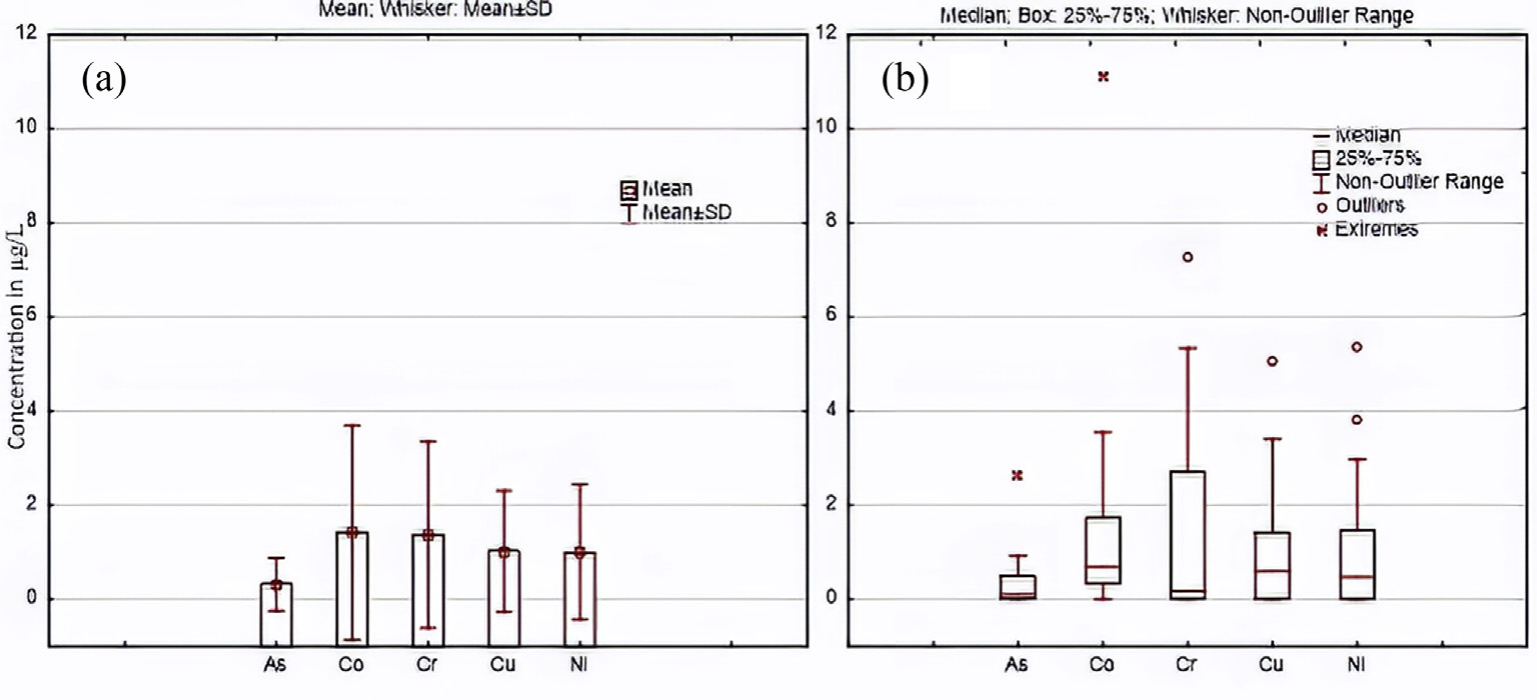
Illustration of (a) regular (bar plot with SD as error bars) and (b) preferred (boxplot) visualisation of skewed data.

Figure 4 illustrates the visualisation of descriptive statistics of water quality data. Bar plots with error bars (a) and boxplot (b) were constructed using the same scale to showcase the suitability of the boxplot already explained above. Meanwhile, the data distribution was positively skewed; thus, boxplot, as can be visualised is a better choice showing the median, percentiles, outlying values, and extreme values in a single plot. It is also shown in Figure 4a that the whiskers (representing ‘±SD’) extended into negative, which is not intuitive and further buttresses the inappropriateness of using error bars to represent the variability of data in this instance.

#### 3.3.2 Visualisation of preferred approaches to CA and inferential statistics

**Figure 5:**
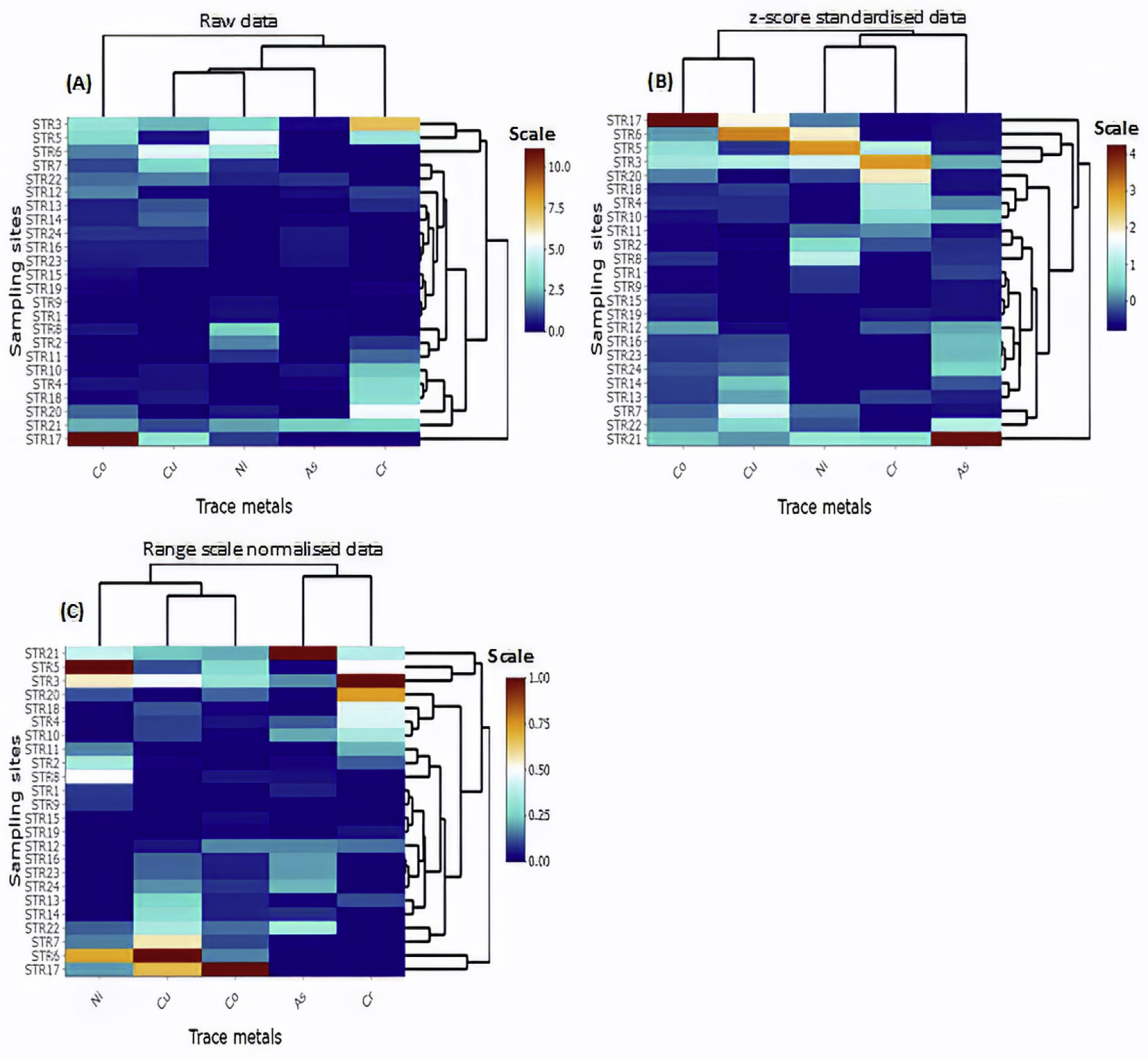
Simulated heatmaps of (a) raw, (b) z-score standardized, and (c) range scale normalised data for cluster analysis.

Part of this sub-subsection is dedicated to visually showing the cluster results of raw skewed data and those resulting from various standardisation methods. This simulation is made to buttress the need for adequate knowledge of the right statistics to be used without resulting in misclassification of variables. The dataset described in section 3.3 was used here. Figure 5 shows heatmaps of different clusters for the trace metals depending on the data used. Based on Figure 5, it could be inferred that the reviewed articles that performed cluster analysis without any form of standardisation would have had a different classification should their data have been standardized or normalised, which is the preferred approach. That means that even those that performed z-score standardisation (seen in almost all the reviewed articles that performed standardisation) but their data did not have an approximately normal distribution might still have wrongly grouped the variables. For instance, clustering the metals in the raw skewed data in Figure 5a, Cu and Ni belonged to the same cluster while Co was in an entirely separate cluster group. Conversely, the cluster analysis of the normalised data (which is preferred for skewed data) put Cu and Co in a cluster instead, but Ni was seen in another cluster, yet in the same cluster group with Cu and Co (Figure 5c). In a hierarchical cluster diagram below shown as heatmaps, different similarity measures can be used including complete linkage, average linkage, and Ward's linkage, which may result in different clusters. However, proper water quality data standardisation or normalisation is required, irrespective of the similarity measure used and depending on the properties of the data.

**Figure 6:**
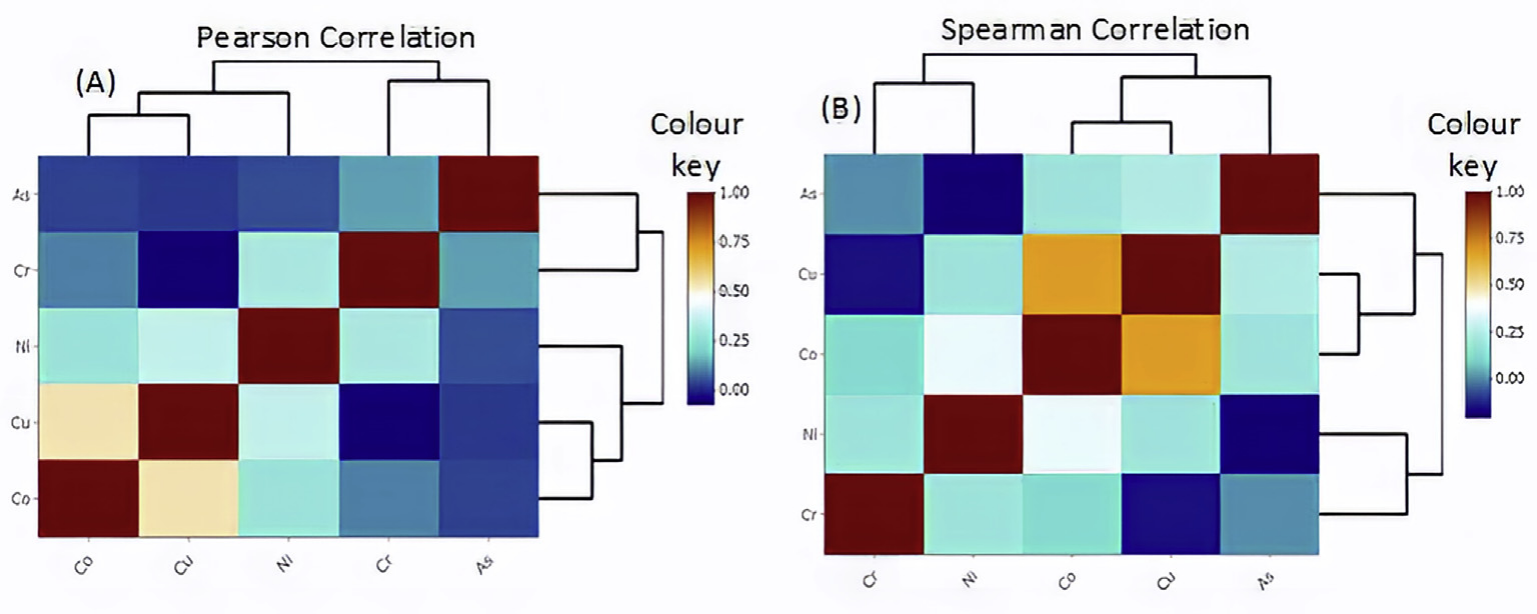
Simulated heatmaps of (a) Pearson correlation, and (b) Spearman correlation.

Furthermore, using heatmaps of correlated variables, we also show that the articles that had small sample sizes and did not transform their data, which might have violated the distributional assumption, possibly had false correlations by employing Pearson correlation method. The implication is that the strong correlation they reported for some variable pairs was indeed weak or not correlated and vice versa. For such skewed data, such anomaly could be averted by transforming the data or using a suitable nonparametric test, in this case, Spearman. Using the simulated dataset, log-transformation was not possible since some of the metals have zero concentrations. Therefore, using heatmaps, Spearman method was shown to possess more power in showing the variable pairs that were significantly correlated. For instance, the Co–Cu pair with a correlation coefficient of ≈0.5 when the Pearson method was applied had an increased coefficient of ≈0.7 when the Spearman method was used (Figure 6). Moreover, it is noteworthy that the strength of the correlation coefficient is not the reason for selecting the Spearman method; instead, it is a distribution-free method suitable for small-size skewed data.

**Figure 7:**
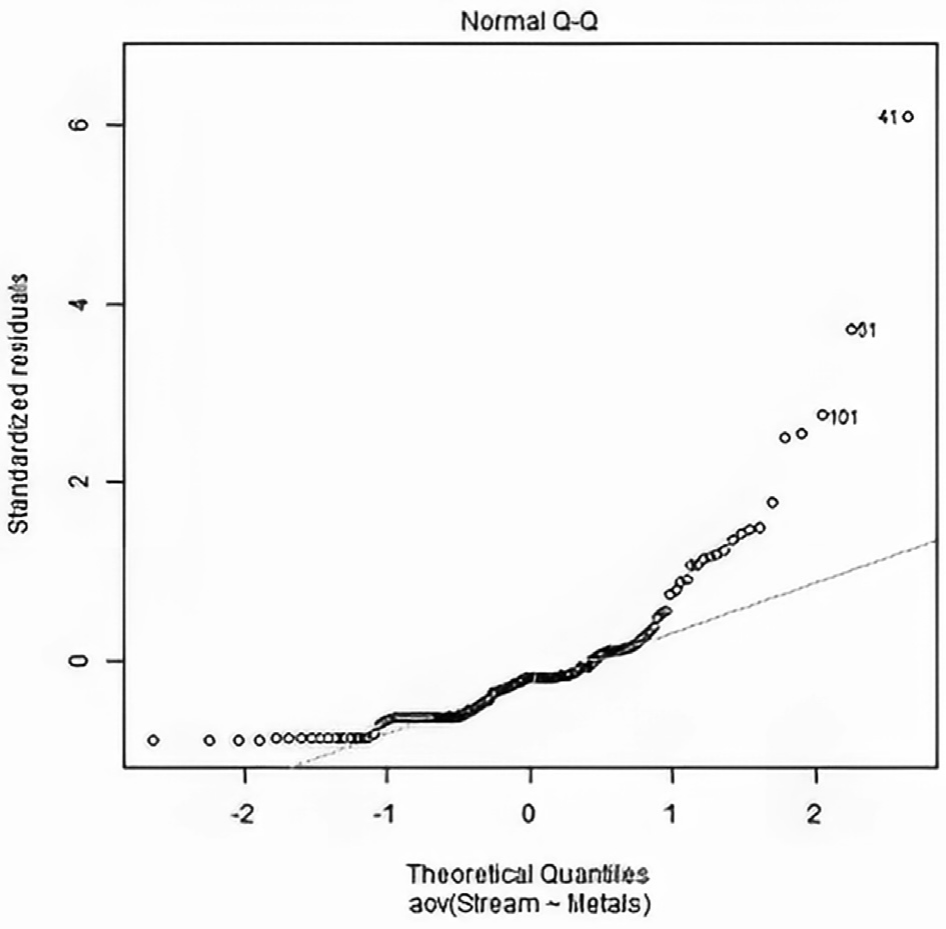
Simulated Q-Q plot of the residuals against the theoretical quantiles.

In comparing group means using ANOVA, for instance, the distribution of the residuals should be approximately normal before using it (Sub-section 3.2.3.1); otherwise, Kruskal-Wallis rank test may be used. The dataset described earlier (Section 3.3) was used to simulate this ‘between-group's means' test. Firstly, it was possible to test for the normality of the residuals through statistical (Shapiro-Wilk test), and visual (normal Q-Q plot) tests in R. The p-value obtained (Shapiro-Wilk normality test: W = 0.72833, p-value = 1.319e-13) was <0.05. The Q-Q plot in Figure 7 corroborates the p-value of the Shapiro-Wilk test, indicating non-normal distribution of the residuals. The deviation of the Q-Q plot from the straight line further shows the skewness of the residuals. All these suggested that the dataset's preferred approach for testing “between-group's means” was the distribution-free Kruskal-Wallis test. Lastly, the p-value of this non-parametric tests (Kruskal-Wallis chi-squared = 7.7416, df = 4, p-value = 0.1015) does not shows enough evidence to reject the null hypothesis. Thus, we conclude that there is no significant difference among the mean concentrations of the trace metals in the stream samples.

#### 3.3.3 Reporting of rounded zeros

Several reporting approaches for rounded zeros were used in the reviewed articles (Supplementary Table S3). Some papers presented them as ‘zeros’, ‘ND’ (not detected), ‘<MDL’ (less than method detection limit), ‘<IDL’ (less than instrument detection limit), <LoQ (less than limit of quantitation) and ‘<DL’ (less than detection limit); while the others did not provide tables to showcase how rounded zeros were presented. Although it may be true that ascertaining the detection or quantitation limits of most volumetric methods of acquiring water quality data presents some difficulties, researchers should endeavour to determine these limits, more especially for instrumental methods. Therefore, it is inappropriate not to perform these method performance evaluation procedures (and not to report them in scientific articles) but report concentrations of variables as “zero” or “ND”. However, if “ND” is written in tables for individual variables, their reporting limits (or at least the equipment detection limit) should be given. Additionally, while it is not uncommon to report values as less than the equipment detection limit, we suggest that reporting limits (such as LoQ or MDL) are used, which are more robust because their determination is performed with higher levels of certainty.

### 3.4 Comparison with some published papers from other countries

For physicochemical parameters, we reviewed about 13 scholarly articles on water quality assessment studies in other countries, including China, India, Nepal, Sri Lanka, Bangladesh, Angola, Ghana, and Taiwan. The result in Table S4 shows that non-reporting of vital steps in data analysis and inappropriate statistics are common in many regions. For example, some articles used boxplot to present their descriptive statistics as a result of obvious skewness of their data, but at the same time tabulated mean and standard deviation (Chai et al., 2021; Koliyabandara et al., 2020; Liu et al., 2021). The preferred reporting of non-normally distributed data was observed in an article where boxplot, and median and range (in a summary table) were used (Wątor and Zdechlik, 2021). Reporting of skewed data with mean ± SD was also identified in some of these articles. Still, we hope that this article and other related articles would be a reference guide for appropriate usage of these statistics. Additionally, some papers (about 46%) standardized (z-score standardisation) their data before performing statistical analysis such as PCA and CA (Table S4), which might not have sufficed for highly skewed data (see section 3.3.2). Generally, the trend in statistical data analysis and reporting observed in the reviewed papers strongly buttress the necessity for the continuous promotion of the correct application of statistical data analysis and reporting of water quality data.

### 3.5 Alternative approaches to commonly used water quality assessment methods

From this literature analysis, we could say that the traditional statistical methods are not in any way less of methods for water quality data; thus, we earnestly advocate their adequate knowledge and appropriate usage. Yet, alternative approaches are not unnecessary as they would at least add varieties to available statistical methods. For instance, performing statistical analysis in environments such as MATLAB and R are fast and add several enhancements to the graphical presentation of data compared to the output of standard software. Moreover, another area worth considering in water quality assessment in Nigeria is artificial intelligence (AI) and the use of online instrumentation.

#### 3.5.1 Machine learning and deep learning methods for water quality assessment

Machine learning (ML) could be seen as a field of artificial intelligence, which involves algorithms that allow computer systems to deduce patterns from data (Hosseini et al., 2020). Alternatively, it is a process designed for a machine to practically learn from an input dataset, develop a structure, and improves it through the data for a specific purpose. Deep learning (DL) is a subfield of machine learning, designed to allow computational models with multiple hidden processing layers to learn data representations with numerous echelons of abstraction. Currently, research is being intensified in the use of these soft computing techniques in water research as well (Barzegar et al., 2017; Hosseini et al., 2020; Zhou et al., 2020). Although traditional models have been used for this purpose, the complexity in hydrogeochemical processes of different water systems requires more advanced techniques (Zou et al., 2020). Therefore, these artificial intelligent models have become more adequate alternatives to ensure adequate water quality assessment and prediction. Incessant improvements have been made in this regard, going from standalone to hybrid architectures (Rajaee et al., 2020), which have been proven to offer better predictive performance by utilizing a feature optimization or a data preprocessing method. This area is relatively new in Nigeria and has not been fully utilized, at least not in water quality research. Therefore, we reviewed the commonly used machine and deep learning methods to find adequate models that will be suitable for the type and size of data being generated in water quality assessment in Nigeria. Consequently, we urge environmental scientists in Nigeria to develop the capability and take advantage of these cost-effective yet accurate and time-saving techniques in assessing water quality.

##### 3.5.1.1 Machine learning methods

The need to develop low-cost and efficient methods for processing huge amounts of linear and non-linear data led to the emergence of ML and DL (Najah Ahmed et al., 2019). Several research works have employed ML for assessing and predicting different water quality variables using various model architectures. Some widely used ML models include Artificial Neural Networks (ANN), Support Vector Machine (SVM), and Decision Tree (DT) (Kim et al., 2014).

Artificial neural network (ANN) has turned out to be a widely employed ML tool, not only in fields of medicine and ecology (Gredell et al., 2019), but also in quality management of various water resources (Khan and See, 2016; Najah Ahmed et al., 2019). ANN is believed to imitate the nervous system networks of the human brain (Khan and See, 2016; Haghiabi et al., 2018); there is a transmission of input signal in a forward direction, from one layer to the next through a network of neurons. This process is known as feed forward propagation because the weights, *w* assigned to each interlayer link are multiplied by the input and the resulting value proceeds forward to the next layer until it gets to the output layer (Khan and See, 2016). Furthermore, in another process called recurrent or feedback propagation, the signal can flow in forward and reverse directions through a specific part or the entire network. In recent times, ANN has been used in other countries to estimate and predict various water quality variables such as nitrate, pH, alkalinity, turbidity, *E. coli*, dissolved oxygen, chlorophyll-a, Ca^2+^ and Mg^2+^ (Al-Mukhtar and Al-Yaseen, 2019; Chou et al., 2018; Mamun et al., 2020; Mohammed et al., 2018). Hence, such studies in Nigeria would ensure the sustainability of water resources through an efficient monitoring system.

It is noteworthy that ANN provides accurate predictions irrespective of measurement error, does not demand the knowledge of the mathematical forms of the association between the input variables and their corresponding outputs, and has fast data processing (Farmaki et al., 2010). Conversely, overfitting (i.e., training data too well, leading to failure to reliably predict future variables) can occur as a result of using many weights, and failure to generalise well when limited data are used for training is imminent (Farmaki et al., 2010). Interestingly, a selection of neural network architectures that are based on feed forward propagation namely: Multilayer Perceptron Neural Network (MLP-ANN or MLP) and Radial Basis Function (RBF-ANN) have exhibited satisfactory potentials to overcome some of the challenges of the standalone neural network. For instance, MLP-ANN has the potential to overcome overfitting that is peculiar to ANN (Farmaki et al., 2010), and can handle non-linear data, which is a demerit of a single layer neural network (Solanki et al., 2015). Similarly, RBF-ANN is advantageous due to its reliability and robustness in noisy data, faster network convergence and training, and ability to adequately generalise with lower samples to variables ratio (Huang and Yang, 2020).

Support vector machine (SVM) depends on Vapnik's theory, a statistical learning theory, which uses limited amount of data to achieve a high level of generalisation and prediction accuracy (Barzegar et al., 2017). Additionally, SVM is typified by an effective mechanism for circumventing overfitting, which results in good performance of the model unlike ANN (Haghiabi et al., 2018). Support vector machine has been utilised in conjunction with genetic programming to predict some water quality variables (Joslyn, 2018). It was noted that SVM yielded a high accuracy of 98.4% and 97.9% for temperature and dissolved oxygen, respectively. However, long prediction time seems to mar the benefits derived using SVM (Barzegar et al., 2017).

In decision tree machine learning, each tree node represents an attribute, each branch (link) represents a decision, while each leaf represents an outcome. The design and interpretation of DTs are simple, accurate, and can handle high dimensional data. DTs offer various advantages such as ability to handle mixed data and missing values, resistant to outliers, and have the ability to manage irrelevant inputs; their lack of hidden layer enables better modelling performance (Bui et al., 2020). However, DTs may also suffer the drawback of overfitting.

##### 3.5.1.2 Deep learning methods

Deep learning models can perform multiple nonlinear transformations due to numerous layers of computational units at each layer (Zhou, 2020). A DL method is a representation learning method with various levels of representation derived from combining simple but non-linear segments that each transforms the representation at one level into another higher and more abstract representation level (Lecun et al., 2015). A unique characteristic of DL is that the layers of features are not pre-design by human; instead, they are learnt from data using general-purpose learning procedures (Lecun et al., 2015). Convolutional neural network (CNN) and Recurrent neural network (RNN) are some of the DL architectures. While CNN is dedicated to solving image processing challenges, RNN finds application in modelling sequence data such as audio, text, or time series for predictions (Lecun et al., 2015). Moreover, long short-term memory (LSTM) and gradient recurrent units (GRU) are two specialised RNNs that are designed to compensate for the short-term memory that undermines RNN resulting from the backpropagation algorithm used for its training (Lecun et al., 2015). Amongst other DL models, CNN and LSTM have been used to predict water quality variables such as dissolved oxygen, chlorophyll-a, NH_3_–N, pH, and COD with acceptable accuracy (Barzegar et al., 2020; Zhou, 2020; Zou et al., 2020).

Summarily, the input dataset is primarily an important element in water quality prediction. Considering the above AI models, it may be inferred that, owing to the nature of the data generated in water quality research in Nigeria, we could leverage the various benefits of ML models (not DL models) for the time being. A case in point is that our data collection method is centered on the traditional grab sampling and analysis (laboratory instrumentation), which preclude the acquisition of time series data needed for most DL modelling. Besides, most of the parameters of interest for the preliminary water quality evaluation can be accurately estimated and predicted using ML models. Also, the relatively small size of the data generated using laboratory instrumentation, as seen in the reviewed articles, warrants the application of ML, particularly ANN and SVM that can achieve a high level of generalisation and prediction accuracy using a limited amount of data (Barzegar et al., 2017).

#### 3.5.2 Water quality assessment using online instrumentation

Water quality assessment is gradually shifting from traditional grab sampling and analysis to online instrumentation. As observed from this review, our data collection method was mainly laboratory instrumentation; thus, it would be desirable to take advantage of online instrumentation. Water quality data have been reportedly collected from different study locations using automated sensors (Zhou, 2020). Although it has shortcomings such as ‘missing data’, methods have been put in place to either fill or augment the missed data (Zou et al., 2020). Monitoring water quality with online instruments has been admitted as dependable and low-cost, taking care of low-frequency monitoring that typifies grab sampling with its many drawbacks. These include the high cost of analysis, the time lag in collecting data, and the difficulty of accounting for scenarios that transpire during sampling intervals (Castrillo and García, 2020). For microbiological assessment, researchers are developing biosensors that aim to provide almost real-time results of the microbial parameters of interest (Tatari et al., 2016). This technology will help overcome the major drawbacks in conventional analyses, including the days-long incubation time required to provide results and the laborious processes involved (Tatari et al., 2016). These systems are especially critical in water quality monitoring, where a rapid response to potential contamination events is necessary to protect public health (Lopez-Roldan et al., 2013). Optical online bacteria sensors that function within a 10-minute time resolution are currently available (Højris et al., 2016). They are based on the optical properties of the water sample and the analytes (including the microorganisms) contained in it (Lopez-Roldan et al., 2013).

Tatari and co-workers surveyed some of the biological sensor technologies that were available in the market at that time (which had potential or were being validated for use for drinking water). They classified these technologies into three categories, namely: (a) those that detected specific indicator organisms (enzyme activity), (b) those that assayed for the total bacteria concentration, and (c) those that assayed for the total bacteria activity (Tatari et al., 2016). These systems can either detect the presence of thermotolerant coliforms (and specifically *E. coli*) or estimate the density of the total number of bacterial cells in a sample of water and does so in rapid time (Lopez-Roldan et al., 2013; Tatari et al., 2016).

### 3.6 Areas of deficiency in water quality research in Nigeria

The implementation of appropriate data analysis and reporting for water quality data will undoubtedly boost the researcher's confidence in Nigeria and bring out the desired advancement in water research. Additionally, funding and collaboration could help improve not only research on water quality but also environmental science research in general, but these areas are defective. Firstly, funding is needed for any meaningful research to be carried out, but in developing countries such as Nigeria, researchers are almost always financing their research. Although, this could be partly blamed on researchers who should come up with quality proposals worthy of financing by local or international funding agencies. However, the pitiable state of science laboratories coupled with dwindling power supply, persistent lecturers’ strike, and non-payment of salaries are all contributors to poor research output in Nigeria. To further buttress the above points, only 22.8% (28 of 123) of the reviewed articles received funding from either local or international funding bodies (Table S2). Moreover, 10 out of the 28 articles were locally funded by bodies such as Tertiary Education Trust Fund (TETFund), Niger Delta Development Commission (NDDC), and Petroleum Technology Development Fund (PTDF) in Nigeria. The authors also observed that the studies that received funding had elaborate studies, consequently publishing their works in relatively high impact journals.

Last but certainly not least is the collaboration among researchers in different fields, local institutions, and foreign institutions. It is obvious what collaboration can do in research. Research groups can collaborate with others in the same or other institutions, leverage their knowledge or equipment, form formidable research allies, and produce quality research output. Indeed, this is not the case in most developing countries such as Nigeria, at least not in water quality research. It was gathered from the reviewed papers that approximately 34.1% of the studies were carried out by researchers in a single department in an institution (Table S2). A case in point is that a single department may not be able to produce comprehensive research for water quality, especially when all the parameters are involved. Furthermore, the authors observed that most departments undertook studies outside their field without a report of consulting a researcher in the other departments. For instance, a researcher in the Physics Department handling physicochemical parameters, while a researcher in the Chemistry Department determining microbiological parameters without any contribution from a microbiologist. Overall, having also reviewed other papers from the developed countries, the authors, therefore, opine that collaboration is necessary for boosting water quality research in Nigeria.

## 4 Conclusion

This article has provided instances in the Nigerian context (but applicable in other countries) where analysis and reporting of water quality data could be misleading; thus, should be avoided. Notwithstanding, preferred analysis, numerical reporting, and visualisation of water quality data were illustrated for use by researchers. Obviously, it may not be imprecise to say that the issues raised in this review are not only peculiar to water research but also to other environmental studies in Nigeria and other countries. Therefore, using a part of the dataset from a previous study was necessitated to simulate visuals and heatmaps showing various preferred approaches to presenting descriptive and some inferential statistics of water quality data. More so, researchers are encouraged to leverage the versatility of artificial intelligence in the assessment and prediction of water quality. Moreover, the type and size of data that can be generated through laboratory experimentation are shown in this review. Thus, ML models, especially ANN and SVM that can achieve a high level of generalisation and prediction accuracy using limited amount of data, are the most appropriate AI architectures to start with. This literature survey conveys an important message to experts, students, researchers, government administrators, and the public about existing knowledge and proffers solutions where there were drifts. Therefore, if subsequent water quality studies in Nigeria and other countries would close the gaps revealed in this literature analysis about data collection, analysis, and reporting, the inferences from water research studies would serve as a veritable tool for the protecting water resources.

## Declarations

### Author contribution statement

All authors listed have significantly contributed to the development and the writing of this article.

### Funding statement

This research did not receive any specific grant from funding agencies in the public, commercial, or not-for-profit sectors.

### Data availability statement

Data will be made available on request.

### Declaration of interests statement

The authors declare no conflict of interest.

### Additional information

No additional information is available for this paper.
