# Supplementary material for: An appraisal of data collection, analysis, and reporting adopted for water quality assessment: A case of Nigeria water quality research
Source: Heliyon. 2021 Sep 6;7(9):e07950. doi: 10.1016/j.heliyon.2021.e07950 (PMC8450204; doi:10.1016/j.heliyon.2021.e07950)
Supplement: Supplementary material [file mmc1.docx]

**A review of data collection, analysis and reporting adopted for water quality assessment:** **A case of Nigeria water quality research**

Ugochukwu Ewuzie^1*^; Nnaemeka O. Aku^2,3^; Stephen U. Nwankpa^4^

^1^Analytical/Environmental Unit, Department of Pure and Industrial Chemistry, Abia State University, Nigeria.

^2^Medical Microbiology Unit, Department of Microbiology, University of Nigeria, Nsukka, Nigeria

^3^Public Health Unit, Department of Community Medicine, University of Nigeria, Enugu Campus, Nigeria

^4^College of Pharmacy, Roseman University of Health Sciences, South Jordan Utah, USA

**SUPPLEMENTARY TABLES**

Table S1. Description of the journals for the reviewed papers

| **Journal** | **2019 IF or SJR** | **Quartile No** | **No of Papers** | **Percentage of papers** |
| --- | --- | --- | --- | --- |
| African Journal of Aquatic Science | 0.778 | **Q3** | 2 | 1.6% |
| Annals of African Medicine | 0.24 | **Q3** | 1 | 0.8% |
| Ain Shams Engineering Journal | 1.949 | **Q1** | 1 | 0.8% |
| Archives of Environmental Contamination and Toxicology | 2.400 | **Q2** | 1 | 0.8% |
| Archives of Environmental & Occupational Health | 1.180 | **Q2** | 2 | 1.6% |
| BMC Research Notes | 0.60 | **Q2** | 1 | 0.8% |
| Bulletin of Environmental Contamination and Toxicology | 1.657 | **Q2** | 2 | 1.6% |
| Chemistry and Ecology | 1.400 | **Q2** | 1 | 0.8% |
| Chemosphere | 5.778 | **Q1** | 5 | 4.1% |
| Ecotoxicology and Environmental Safety | 4.872 | **Q1** | 1 | 0.8% |
| Emerging Contaminants | 1.99 | **Q1** | 1 | 0.8% |
| Environmental Chemistry | 1.91 | **Q2** | 1 | 0.8% |
| Environmental Earth Science | 2.180 | **Q2** | 2 | 1.6% |
| Environmental Monitoring and Assessment | 1.903 | **Q2** | 30 | 24.4% |
| Environmental Nanotechnology, Monitoring and Management | 0.84 | **Q1** | 2 | 1.6% |
| Environmental Science and Pollution Research | 3.056 | **Q2** | 3 | 2.4% |
| Environmental Technology and Innovation | 3.356 | **Q1** | 2 | 1.6% |
| Environmental Management | 2.561 | **Q1** | 1 | 0.8% |
| Environmental Pollution | 6.792 | **Q1** | 1 | 0.8% |
| Environmental Research, Engineering and Management | 0.15 | **Q4** | 1 | 0.8% |
| Environmental Geochemistry and Health | 3.472 | **Q1** | 1 | 0.8% |
| Exposure and Health | 4.762 | **Q1** | 2 | 1.6% |
| Groundwater for Sustainable Development | 0.87 | **Q1** | 6 | 4.9% |
| Heliyon | 0.43 | **Q1** | 6 | 4.9% |
| International Journal of Environmental Research and Public Health | 2.849 | **Q2** | 2 | 1.6% |
| International Biodeterioration & Biodegradation | 4.074 | **Q1** | 1 | 0.8% |
| International Journal of Environmental Health Research | 1.916 | **Q3** | 1 | 0.8% |
| Journal of African Earth Sciences | 1.603 | **Q2** | 5 | 4.1% |
| Journal of Environmental Chemical Engineering | 0.93 | **Q1** | 2 | 1.6% |
| Journal of Environmental Management | 5.647 | **Q1** | 2 | 1.6% |
| Journal of Environmental Science and Health, Part A | 1.724 | **Q2** | 2 | 1.6% |
| Journal of Environmental and Public Health | 0.71 | **Q2** | 1 | 0.8% |
| Journal of Food Composition and Analysis | 3.721 | **Q1** | 1 | 0.8% |
| Journal of Health and Pollution | 0.25 | **Q3** | 5 | 4.1% |
| Journal of King Saud University-Science | 3.819 | **Q1** | 2 | 1.6% |
| Journal of Water and Health | 1.349 | **Q2** | 1 | 0.8% |
| Marine Pollution Bulletin | 4.049 | **Q1** | 1 | 0.8% |
| Microchemical Journal | 3.594 | **Q2** | 1 | 0.8% |
| Pakistan Journal of Science and Industrial Research Part A: Physical Sciences | 0.11 | **Q4** | 1 | 0.8% |
| Pakistan Journal of Biological Sciences | 0.83 | **Q3** | 1 | 0.8% |
| Physics and Chemistry of the Earth | 2.308 | **Q2** | 1 | 0.8% |
| Progress in Physical Geography | 3.488 | **Q1** | 1 | 0.8% |
| Public Health | 1.774 | **Q2** | 2 | 1.6% |
| Radiation Physics and Chemistry | 2.226 | **Q2** | 2 | 1.6% |
| Regional Studies in Marine Sciences | 1.183 | **Q2** | 1 | 0.8% |
| Revista de Biologia Tropical | 0.515 | **Q2** | 1 | 0.8% |
| Toxicological and Environmental Chemistry | 1.149 | **Q3** | 1 | 0.8% |
| Toxicology Report | 0.91 | **Q1** | 4 | 3.3% |
| Turkish Journal of Fisheries and Aquatic Sciences | 0.869 | **Q3** | 2 | 0.8% |
| Water and Environment Journal | 1.426 | **Q2** | 1 | 0.8% |
| Water Resources and Industry | 1.02 | **Q1** | 1 | 0.8% |
| Water, Air and Soil Pollution | 1.900 | **Q2** | 1 | 0.8% |
|  |  | **Total** | 123 | 100.0% |

Table S2. Details of the reviewed papers regarding funding and collaboration

| **S/No** | **Reference** | **Journal** | | | **Parameter** | | **Funding** | | **Department/ Institute/ Research Centre** | | **Collaboration** | |
| --- | --- | --- | --- | --- | --- | --- | --- | --- | --- | --- | --- | --- |
| **Groundwater** | |  | | |  | |  | |  | |  | |
| 1 | (Adiat et al., 2020) | Environ Monit Assess | | | P/C/Metal | | Yes | | Applied Geophysics | | 1 Uni Local | |
| 2 | (Bamigboye et al., 2020) | Groundwater for Sustainable Development | | | P/C | | No | | Pure & Applied Bio; Ecology & Resource Mgt | | 1 Uni Local, 1 Uni Int'l | |
| 3 | (Egbueri et al., 2020) | Environ Monit Assess | | | Metal | | No | | Geology | | 3 Uni Local | |
| 4 | (Egbueri, 2020) | Groundwater for Sustainable Development | | | Metal | | No | | Geology | | 1 Uni Local | |
| 5 | (Eyankware et al., 2020) | Groundwater for Sustainable Development | | | P/C/Metal | | No | | Geology; Steel Raw Material Exploration | | 2 Uni Local, 1 centre | |
| 6 | (Gbadebo, 2020) | Envrion Geochem Health | | | P/C/Metal | | No | | Environ Mgt & Toxicol | | 1 Uni Local | |
| 7 | (Jagaba et al., 2020) | Ain Shams Engineering Journal | | | P/C/Metal | | Yes | | Civil Engineering; Institute of Energy Infrac | | 1 Uni Local, 3 Uni Int'l | |
| 8 | (Okunola et al., 2020) | Heliyon | | | Metal/RadE | | No | | Chemistry; Oncology; Energy Res & Training | | 1 Uni Local, 2 Centres | |
| 9 | (Owamah, 2020) | Groundwater for Sustainable Development | | | P/C/Metal | | Yes | | Civil Engineering | | 1 Uni Local | |
| 10 | (Shu’aibu et al., 2020) | Radiation Physics and Chemistry | | | RadE | | No | | Physics | | 2 Uni Local, 1 Uni Int'l | |
| 11 | (Talabi et al., 2020) | Groundwater for Sustainable Development | | | P/C/Metal | | No | | Geology; Science Lab Technology | | 1 Uni Local | |
| 12 | (Ugwoke & Waziri, 2020) | Journal of African Earth Sciences | | | Metal | | No | | Geology | | 2 Uni Local | |
| 13 | (Zacchaeus et al., 2020) | Heliyon | | | P/C/Metal | | Yes | | Chemistry; Physics; Environ Mgt & Toxicol | | 3 Uni Local, 1 Uni Int'l | |
| 14 | (Adiat et al., 2019) | Heliyon | | | P/C/Metal | | No | | Applied Geophysics | | 1 Uni Local | |
| 15 | (Aganbi et al., 2019) | Toxicological Report | | | PCBs | | Yes | | Chemistry; Chemistry & Physics; Biochem | | 1 Uni Local, 1 Uni Int'l | |
| 16 | (Aromolaran et al., 2019) | Environ Monit Assess | | | P/C/Metal/Micro | | No | | Biological Sci; Microbiology; Geological Sci; Physics &Chemical Sci | | 4 Uni Local, 1 Uni Int'l | |
| 17 | (Ekere et al., 2019) | Environ Monit Assess | | | P/C | | No | | Chemistry | | 1 Uni Local | |
| 18 | (Ibrahim et al., 2019) | Groundwater for Sustainable Development | | | P/C/Metal | | No | | Civil Engineering; Geology & Mineral Sci; Groundwater Studies | | 1 Uni Local, 1 Uni Int'l | |
| 19 | (Olufemi V. Omonona et al., 2019) | Environ Monit Assess | | | P/C/Metal | | No | | Geology; Mathematics & Statistics; Physics & Geology | | 5 Uni Local | |
| 20 | (Onwuka et al., 2019) | Journal of African Earth Sciences | | | Metal/ REE | | No | | Geology; Physics & Geology | | 2 Uni Local | |
| 21 | (Owoyemi et al., 2019) | Environ Monit Assess | | | P/C/Metal | | No | | Petroleum Engineering; Geology; Physics & Geology | | 3 Uni Local | |
| 22 | (Turajo et al., 2019) | Environ Science Pollution and Research | | | P/C/Metal | | No | | Engineering | | 3 Uni Local | |
| 23 | (Ubuoh et al., 2019) | Environ Tech and Innovation | | | Metal | | No | | Environ Mgt & Toxicol; Engineering | | 1 Uni Local | |
| 24 | (P. G. C. Emenike et al., 2018) | Environ Monit Assess | | | P/C/Metal | | No | | Civil Engineering | | 2 Uni Local | |
| 25 | (C. P. G. Emenike et al., 2018) | Ecotoxicological & Environ Safety | | | P/C/Metal | | No | | Civil Engineering; Water, Energy & Environ | | 1 Uni Local, 1 Uni Int'l | |
| 26 | (Odipe et al., 2018) | Journal of Health and Pollution | | | P/C/Metal | | No | | Geology; Environ Health Sci; Ministry of Water Resources | | 2 Uni Local, 1 centre | |
| 27 | (Ukah et al., 2018) | Environ Monit Assess | | | P/C/Metal/ Micro | | No | | Geology | | 1 Uni Local | |
| 28 | (A. S. Adekunle et al., 2017) | Toxicological Report | | | PAHs | | No | | Chemistry | | 2 Uni Local, 1 Uni Int'l | |
| 29 | (Ejike et al., 2017) | Environ Science Pollution and Research | | | Metal | | No | | Biochemistry | | 3 Uni Local | |
| 30 | (Omaka et al., 2017) | Environ Earth Science | | | P/C/Metal | | No | | Physics & Geology; Chemistry; Science Lab Tech | | 3 Uni Local | |
| 31 | (Sawyerr et al., 2017) | Journal of Health and Pollution | | | P/C/Metal | | No | | Environ Mgt & Toxicol; Environ Health Sci, Ecological & Environ Res | | 2 Uni Local | |
| 32 | (Stephen et al., 2017) | Journal of African Earth Sciences | | | P/C/Metal | | No | | Geology & Exploration Geophysics | | 1 Uni Local | |
| 33 | (Aboyeji & Eigbokhan, 2016) | Journal of Environmental Management | | | P/C/Metal | | No | | Centre for Aerospace surveys | | 1 centre Local | |
| 34 | (Omaka et al., 2016) | Pakistan J. Sci. Ind. Res Part A. Physical Sci | | | P/C/Metal | | No | | Chemistry | | 3 Uni Local | |
| 35 | (Sojobi, 2016) | Environ Monit Assess | | | P/C/Metal | | No | | Civil Engineering | | 1 Uni Local | |
| 36 | (Aboh et al., 2015a) | Annals of African Medicine | | | Microbiological | | No | | Textile Sci & Tech; Microbiology | | 1 Uni Local | |
| 37 | (Ayedun et al., 2015) | Environ Monit Assess | | | P/C/Metal | | No | | Environ Mgt & Toxicol; Water Resources Mgt; Chemical Sciences | | 2 Uni Local | |
| 38 | (Maxwell et al., 2015) | Radiation Physics and Chemistry | | | RadE | | Yes | | Physics; Energy Res & Develop | | 1 Uni Local, 2 Uni Int'l | |
| 39 | (Akoteyon, 2014) | Environ Research, Engineering and Management | | | P/C/Metal | | No | | Geography and Planning | | 1 Uni Local | |
| 40 | (Dahunsi et al., 2014) | Exposure and Health | | | P/C/Metal/Micro | | No | | Pure & Applied Bio; Biological Sci.; Civil Engineering | | 3 Uni Local | |
| 41 | (O. V. Omonona et al., 2014) | Environ Monit Assess | | | P/C/Metal | | No | | Geology | | 1 Uni Local | |
| 42 | (Zume, 2011) | Journal of Water and Health | | | C/Phenol/Formaldehyde | | Yes | | Geography and Earth Sci | | 1 Uni Int'l | |
| 43 | (Akinyemi et al., 2011) | Public Health | | | Microbiological | | No | | Engineering & Environ Sci.; Water Resources Mgt & Agric Meteo | | 1 Uni Local | |
| 44 | (O. O. Odukoya et al., 2010) | Environ Monit Assess | | | Metal/Phenol | | No | | Chemistry | | 2 Uni Local | |
| 45 | (I. M. Adekunle et al., 2007) | Int'l Journal of Environ Research & Public Health | | | P/C/Metal/Micro | | No | | Environ Mgt & Toxicol; Soil Sciences & Land Mgt | | 1 Uni Local | |
| 46 | (Ejechi et al., 2007) | Environ Monit Assess | | | P/C | | Yes | | Botany & Microbiology; Geology; Chemistry; Natural & Applied Sci. | | 1 Uni Local | |
| 47 | (Aremu et al., 2002) | Int'l Journal of Environ & Health Research | | | C/Metal | | No | | Medicine; Medical Environmentology | | 1 Uni Local, 1 Uni Int'l | |
| 48 | (Ikem et al., 2002) | Water, Air and Soil Pollution | | | P/C/Metal | | No | | Chemistry; Engineering; Public Health & Preventive Medicine | | 1 Uni Local, 1 Uni Int'l | |
| **Surface water** | |  | | |  | |  | |  | |  | |
| 49 | (Alum & Okoye, 2020) | Environ Monit Assess | | | P/C/metal/Micro | | No | | Chemistry | | 1 Uni Local | |
| 50 | (P. G. C. Emenike et al., 2020) | Chemosphere | | | P/C/metal | | No | | Engineering; Water Sci. Institute; Exact & Tech Sci. | | 2 Uni Local, 4 Uni Int'l | |
| 51 | (Ihunwo et al., 2020) | Marine Pollution Bulletin | | | Metal | | No | | Chemistry; Biochemistry & Chemistry | | 2 Uni Local | |
| 52 | (Nganje et al., 2020) | Journal of African Earth Sciences | | | Metal | | Yes | | Geology; School of Sci, & Sports | | 2 Uni Local, 1 Uni Int'l | |
| 53 | (Ogunbanwo et al., 2020) | Environ Chemistry | | | Pharmaceuticals | | Yes | | Eco-chemistry, Environment; Geography | | 3 Uni Int'l, 1 Agency, 1 Uni Local | |
| 54 | (Chukwuka et al., 2019) | Chemosphere | | | Pesticides | | No | | Entomology & Oncology; Environ Mgt & Toxicol; NESREA | | 1 Uni Local, 1 Uni Int'l, 1 centre | |
| 55 | (Daramola et al., 2019) | Heliyon | | | P/C/metal | | Yes | | Geology; Social Sci. & Humanities | | 1 Uni Int'l | |
| 56 | (Ezemonye et al., 2019) | Toxicological Report | | | P/C/metal | | No | | Energy & Environ; Environ Mgt & Toxicol; Animal & Environ Bio; Lab of Toxicol | | 2 Uni Local, 1 Uni Int'l | |
| 57 | (Ibe et al., 2019) | Environ Monit Assess | | | P/C/metal | | No | | Chemistry; Geology; Health Education | | 3 Uni Local | |
| 58 | (Chukwuka et al., 2019) | Chemosphere | | | Pesticides | | No | | Animal & Environ Bio; NESREA; Environ Mgt & Toxicol; Zoology & Entomology | | 3 Uni Local, 2 Uni Int'l | |
| 59 | (Sogbanmu et al., 2019) | Environmental Pollution | | | PAHs | | No | | Zoology; Marine Sci. | | 1 Uni Local | |
| 60 | (Adesiyan et al., 2018) | Journal of Health & Pollution | | | Metal | | No | | Chemistry; Microbiology; Encology & Environ Studies | | 2 Uni Local, 1 Uni Int'l | |
| 61 | (Ayandiran et al., 2018) | Water Resources and Industry | | | P/C/Metal | | No | | Pure & Applied Bio.; Biological Sci | | 2 Uni Local | |
| 62 | (Beshiru et al., 2018) | Environ Monit Assess | | | P/C/Metal | | Yes | | Microbiology; Environ Health Sci | | 3 Uni Local | |
| 63 | (Ibanga et al., 2019) | Regional Studies in Marine Sciences | | | P/C/Metal | | Yes | | Marine Sci; NDDC | | 1 Uni Local, 1 centre Local | |
| 64 | (Ogbeide et al., 2018) | Journal of Environ Management | | | Pesticides | | No | | Animal & Environ Bio; NESREA; Environ Mgt & Toxicol; Zoology & Entomology | | 3 Uni Local, 1 Uni Int'l, 1 centre Local | |
| 65 | (Ololade et al., 2018) | Journal of Environ Chemical Engineering | | | PFOS & PFOA | | Yes | | Chemical Sci; Earth Sci; Chemistry | | 3 Uni Local, 1 Uni Int'l | |
| 66 | (Titilawo et al., 2018) | Chemosphere | | | Metal | | No | | Chemistry; Microbiology; SAMRC | | 3 Uni Local, 1 Uni Int'l | |
| 67 | (Joe-Ukairo & Oni, 2018) | Journal of Health & Pollution | | | P/C/Metal | | No | | Geology; Min of Water Resources | | 1 Uni Local, 1 Agency Local | |
| 68 | (Ifelebuegu et al., 2017) | Environ Monit Assess | | | P/C/metal/BTEX & PAHs | | No | | School of Energy; Environment Centre | | 2 Uni Int'l | |
| 69 | (A. M. Odukoya et al., 2017) | Environ Nanotech, Monit and management | | | Metal | | No | | Physics; Chemistry; Geosciences | | 2 Uni Local | |
| 70 | (Tongo et al., 2017) | Journal of Envrion Chemical Engineering | | | P/C/PAHs | | No | | Animal & Environ Biology | | 1 Uni Local | |
| 71 | (Usese et al., 2017) | Environmental Technology and Innovation | | | Metal | | Yes | | Oceanography & Marine Res; Marine Sci.; GCER; CRC CARE | | 1 Uni Local, 1 Uni Int'l, 1 centre Local | |
| 72 | (Ogunkunle et al., 2016) | Journal of King Saud University-Science | | | P/C/metal | | No | | Plant Biology | | 1 Uni Local | |
| 73 | (Olatunji & Ajay, 2016) | Journal of Health & Pollution | | | P | | No | | Geology | | 1 Uni Local | |
| 74 | (Adamu et al., 2015) | Environ Nanotech, Monit and management | | | P/C/metal | | No | | Geology | | 1 Uni Local | |
| 75 | (Chia & Kwaghe, 2015) | Environ Monit Assess | | | P/C | | No | | Biological Sci; Agriculture | | 1 Uni Local, 1 Uni Int'l | |
| 76 | (Inam et al., 2015) | Bull Environ Contam Toxicol | | | Pharmaceuticals | | Yes | | Chemistry; Microbiology; Min of Environ; IEAEC | | 2 Uni Local, 2 Uni Int'l | |
| 77 | (Vincent-Akpu et al., 2015) | Toxicological and Environmental Chemistry | | | P/C/Metal | | Yes | | Animal & Environ Bio; Biological & Environ Sci; Environ Res Centre | | 1 Uni Local, 2 Uni Int'l | |
| 78 | (Ogwueleka, 2014) | Water and Environ Journal | | | P/C/metal/Micro | | No | | Civil Engineering | | 1 Uni Local | |
| 79 | (Anyanwu et al., 2013) | African Journal of Aquatic Science | | | P/C/metal | | No | | Animal & Environ Bio | | 1 Uni Local | |
| 80 | (Chigor et al., 2012) | Environ Monit Assess | | | P/C/Micro | | No | | Biochemistry & Microbiology; Microbiology; Water Resources & Environ | | 2 Uni Local, 1 Uni Int'l | |
| 81 | (Eneji et al., 2012) | Environ Monit Assess | | | P/C/metal | | No | | Chemistry; Fisheries and Aquaculture | | 2 Uni Local | |
| 82 | (A. Mustapha & Aris, 2012) | Journal of Environ Sci and health, Part A | | | P/C/metal/Micro | | Yes | | Environ Sci. | | 1 Uni Int'l | |
| 83 | (Adediji et al., 2011) | Progress in physical geography | | | P/C/metal | | No | | Geography | | 2 Uni Local | |
| 84 | (Kolawole et al., 2011) | International Journal of Environmental Research and Public Health | | | P/C/Micro | | Yes | | Biochemistry & Microbiology; Microbiology | | 2 Uni Local, 1 Uni Int'l | |
| 85 | (Nduka & Orisakwe, 2011) | Environ Sci Pollut and Res | | | P/C/metal | | No | | Chemistry; Clinical Pharmacy | | 2 Uni Local | |
| 86 | (Adesalu et al., 2010) | Revista de biologia tropical | | | P/C/metal | | No | | Botany &Microbiology | | 1 Uni Local | |
| 87 | (Williams & Benson, 2010) | Environ Monit Assess | | | P/C/Metal | | No | | Chemistry; Atmospheric & Oceanic Sci | | 1 Uni Local, 1 Uni Int'l | |
| 88 | (Achudume, 2009) | Bull Environ Contam Toxicol | | | P/C/metal | | No | | Institute of Ecology & Environ Studies | | 1 Uni Local | |
| 89 | (Ipeaiyeda & Onianwa, 2009) | Chemistry and ecology | | | P/C/metal | | No | | Chemistry | | 1 Uni Local | |
| 90 | (M. K. Mustapha, 2009) | Turkish journal of Fisheries and aquatic sciences | | | P/C | | No | | Zoology | | 1 Uni Local | |
| 91 | (Adesalu & Nwankwo, 2008) | Pakistan Journal of Biological Sciences | | | P/C | | No | | Botany &Microbiology; Marine Sci | | 1 Uni Local | |
| 92 | (M. K. Mustapha, 2008)m | Turkish journal of Fisheries and aquatic sciences | | | P/C | | No | | Zoology | | 1 Uni Local | |
| 93 | (Arimoro et al., 2007a) | Pakistan Journal of Biological Sciences | | | P/C | | No | | Chemistry; Zoology | | 1 Uni Local | |
| 94 | (Arimoro et al., 2007b) | Ecological Indicators | | | P/C | | No | | Chemistry; Zoology | | 1 Uni Local | |
| 95 | (Jaji et al., 2007) | Environ Monit Assess | | | P/C/metal/Micro | | No | | Environ Mgt & Toxicol; Chemical Sci | | 1 Uni Local | |
| 96 | (Igwilo et al., 2006) | Archives of Environ and occupational health | | | P/C/metal | | No | | Applied Biochemistry; Pharmacology | | 1 Uni Local | |
| 97 | (Uzoukwu et al., 2004) | Environmental Management | | | P/C/Metal | | Yes | | Chemistry | | 1 Uni Local | |
| **Surface & Groundwater** | | |  | |  | |  | |  | |  | |
| 98 | (Adesakin et al., 2020) | Heliyon | | | P/C/Micro | | No | | Biology; Zoology; Ecology & Environ Studies | | 2 Uni Local | |
| 99 | (Bello et al., 2020) | Microchemical Journal | | | RadE | | Yes | | Physics; Energy Res & Training | | 2 Uni Local, 1 centre Local | |
| 100 | (Ewuzie et al., 2020) | Chemosphere | | | Metal | | Yes | | Chemistry | | 1 Uni Local, 1 Uni Int'l | |
| 101 | (Ocheli et al., 2020) | Environ Monit Assess | | | P/C/Micro | | No | | Geology; Geological Sci.; Chemical Sci. | | 3 Uni Local | |
| 102 | (Nnorom et al., 2019) | Heliyon | | | P/C/Metal | | No | | Chemistry | | 1 Uni Local | |
| 103 | (Onyekwelu & Aghamelu, 2019) | Environ Monit Assess | | | Phthalates | | No | | Geology & Mining; Physics & Geology | | 2 Uni Local | |
| 104 | (Fakayode & Ogunjobi, 2018) | Int'l Biodeterioration & Biodegradation | | | P/C/Metal/ Micro | | Yes | | Microbiology | | 2 Uni Local | |
| 105 | (Osinowo, 2016) | Journal of African Earth Sciences | | | P/C | | No | | Geology | | 1 Uni Local | |
| 106 | (Igwe et al., 2015) | Environ Earth Sci | | | P/C/metal/Micro | | No | | Geology; Earth Sci; Food Sci & Tech | | 3 Uni Local | |
| 107 | (Edet & Worden, 2009) | Environ Monit Assess | | | P/C/Metal | | Yes | | Geology; Earth & Ocean Sci. | | 1 Uni Local, 1 Uni Int'l | |
| 108 | (Omo-Irabor et al., 2008) | Physics and Chemistry of the Earth | | | P/C/Metal/TPH | | Yes | | Geology; Built & Natural Environ; Urban Water Tech Centre | | 2 Uni Local, 1 Uni Int'l | |
| 109 | (Rim-Rukeh et al., 2007) | Environ Monit Assess | | | P/C/Metal | | No | | Chemistry; integrated Sci. | | 1 Uni Local | |
| 110 | (Olajire & Imeokparia, 2001) | Environ Monit Assess | | | P/C/metal | | No | | Chemistry | | 1 Uni Local | |
| **Surface, Ground & Rainwater** | |  | |  | |  | |  | |  | |  |
| 111 | (Nganje et al., 2015) | Exposure and Health | | | P/C/Metal | | Yes | | Geology; School of Science | | 1 Uni Local, 1 Uni Int'l | |
| **Surface, Ground & Packaged water** | |  | | |  | |  | |  | |  | |
| 112 | (Afonne et al., 2020) | Journal of Environ Sci & Health | | | P/C/Metal | | No | | Pharmacology | | 1 Uni Local | |
| 113 | (Ebele et al., 2020) | Emerging Contaminants | | | Pharmaceuticals | | No | | Chemistry; Environ Health & Risk Mgt | | 1 Uni Local, 2 Uni Int'l | |
| 114 | (Okafor & Ogbonna, 2003) | Journal of Food Composition and Analysis | | | Metal | | No | | Chemical Sci | | 1 Uni Local | |
| **Sachet/bottled water** | |  | | |  | |  | |  | |  | |
| 115 | (Opafola et al., 2020) | Journal of King Saud University – Science | | | P/C/Metal/Micro | | No | | Civil Engineering; CAS China | | 3 Uni Local, 1 Centre Int'l | |
| 116 | (P. G. C. Emenike et al., 2017) | Environ Monit Assess | | | P/C/Metal | | No | | Civil Engineering | | 2 Uni Local | |
| 117 | (Okorie et al., 2015) | Toxicology Reports | | | P/C/Metal | | No | | Chemistry; NRCRI | | 1 Uni Local, 1 Centre Local | |
| 118 | (Igbenegbu & Lamikanra, 2014) | BMC Res notes | | | Microbiological | | No | | Pharmaceutics | | 1 Uni Local | |
| 119 | (Olaoye & Onilude, 2009) | Public Health | | | Microbiological | | Yes | | Botany & Microbiology; Food Sci | | 1 Uni Local, 1 Uni Int'l | |
| 120 | (Orisakwe et al., 2006) | Archives of Environmental & Occupational Health | | | Metal | | No | | Chemistry; Applied Biochemistry; Pharmacology | | 1 Uni Local | |
| **Rainwater** | |  | | |  | |  | |  | |  | |
| 121 | (Imarhiagbe & Osarenotor, 2020) | Environ Monit Assess | | | P/C/Metal/Micro | | No | | Environ Mgt & Toxicol; Applied Environ Bioscience; RWESCK | | 1 Uni Local, 1 Uni Int'l | |
| 122 | (Igbinosa & Aighewi, 2017) | Environ Monit Assess | | | P/C/Metal | | No | | Environ Mgt & Toxicol | | 2 Uni Local | |
| 123 | (Akintola et al., 2016) | Arch Environ Contam Toxicol | | | Metal | | No | | Agricultural & Environ Engr; Chemical Sci; NHRIN | | 2 Uni Local, 1 centre Local | |

“P” is Physical parameter; “C” is Chemical parameter including anion; “Micro” is Microbiological; “RadE” is Radioactive element; “REE” is Rare earth element; “T.Org” is Trace organics; “PFOA” is Perfluorooctanoic acid; “PFOS” is Perfluorooctanesulphonate; “PCBs” is Polychlorinated biphenyls; “PAHs” is Polycyclic aromatic hydrocarbons; Uni is University

Table S3. Overview of the data collection, analysis and reporting approaches for all parameters

| No of samples | Standard protocol for data collection | | | Standardization and transformation | | | | Reporting style for rounded zeros | | Descriptive statistics | | | How mean and SD were reported | | | Normality test | | | Chosen statistics | | Reference |
| --- | --- | --- | --- | --- | --- | --- | --- | --- | --- | --- | --- | --- | --- | --- | --- | --- | --- | --- | --- | --- | --- |
| **Physicochemical parameters** | |  | | |  | |  | | | |  | | |  | | |  |  |  |  |  |
|  |  | | |  | | | |  | |  | | |  | | |  | | |  | |  |
| 25 | APHA, 2005 | | | Not reported | | | | *Not applicable | | Mean, Min, Max, SD | | | Separately | | | Not reported | | | FA, HCA | | Egbueri et al., 2020 |
| 20 | APHA, 2005 | | | Not reported | | | | Zero | | Mean, Min, Max | | | Separately | | | Not reported | | | HCA | | Egbueri, 2020 |
| 124 | ASTM, D1976 | | | Not reported | | | | <MDL | | Mean, SE | | | Mean ± SD | | | Shapiro-Wick | | | ANOVA | | Ewuzie et al., 2020 |
| 29 | APHA, 1980; ASTM, 1982 | | | Not reported | | | | Not applicable | | Individual values | | |  | | | Not reported | | | Correlation | | Ugwoke & Waziri, 2020 |
| 20 | ASTM, D1971-16 | | | Not reported | | | | <MDL | | Med, Mean, Min, Max, SD, SE | | | Separately | | | Not applicable | | | Not reported | | Ihunwo et al., 2020 |
| 25 | Literature | | | Not reported | | | | Zero | | Mean, Min, Max, SD, | | | Separately | | | Not applicable | | | Not reported | | Nganje et al., 2020 |
| 12 | Literature | | | Not reported | | | | Not applicable | | Mean, SD | | | Mean ± SD | | | Not reported | | | Correlation | | Okunola et al., 2020 |
| 122 | Not reported | | | Not reported | | | | Not applicable | | Individual values | | |  | | | Not reported | | | Pearson correlation | | Bamigboye et al., 2020 |
| 10 | Not reported | | | Not reported | | | | ND | | Individual values | | |  | | | Kurtosis | | | Spearman correlation | | Adiat et al., 2020 |
| 20 | APHA, 2540C | | | Not reported | | | | ND | | Individual values | | |  | | | Not reported | | | Pearson correlation | | Afonne et al., 2020 |
| 30 | APHA, 2012 | | | Not reported | | | | Zero | | Mean, Min, Max | | | Separately | | | Not applicable | | | Not reported | | Eyankware et al., 2020 |
| 52 | Literature | | | Not reported | | | | Not applicable | | Mean, Min, Max, SD | | | Separately | | | Not applicable | | | Not reported | | Gbadebo, 2020 |
| 15 | APHA, 3500; 4500; etc. | | | Not reported | | | | Zero | | Mean, Min, Max, SD, SE, V | | | Separately | | | Kurtosis, Skewness | | | ANOVA; correlation | | Jagaba et al., 2020 |
| 240 | APHA, 2012; Literature | | | Not reported | | | | ND | | Mean, Min, Max, SD, | | | Separately | | | Not reported | | | ANOVA; Correlation | | Owamah, 2020 |
| 30 | USEPA, 1983 | | | Not reported | | | | Zero | | Mean, Min, Max, SD, | | | Separately | | | Not applicable | | | Not reported | | Talabi et al., 2020 |
| 80 | APHA, 1998 | | | Not reported | | | | ND | | Individual values | | |  | | | Not applicable | | | Not reported | | Zacchaeus et al., 2020 |
| 72 | Not reported | | | Not reported | | | | Not applicable | | Individual values | | |  | | | Not reported | | | PCA; Correlation | | P.G.C. Emenike et al., 2020 |
| 21 | APHA, 2012; USEPA, 2016 | | | Not reported | | | | <IDL | | Mean, SE | | | Mean ± SE | | | Not reported | | | ANOVA | | Opafola et al., 2020 |
| 24 | APHA 1998 | | | Not reported | | | | Zero | | Mean, SD | | | Mean ± SD | | | Not reported | | | PCA | | Imarhiagbe & Osarenotor, 2020 |
| 54 | APHA, 2012 | | | Not reported | | | | Not applicable | | Mean, Min, Max, SD, | | | Separately | | | Not reported | | | Pearson correlation | | Alum & Okoye, 2020 |
| 72 | APHA, 1995; ASTM, 1990, Literature | | | Not reported | | | | Not applicable | | Mean, V, CV, SD, | | | Separately | | | Not reported | | | Correlation coefficient, t-test, chi-square test | | Ocheli et al., 2020 |
| Not reported | APHA, 2001 | | | Not reported | | | | Not applicable | | Mean, Min, Max, SE | | | Mean ± SE | | | Not reported | | | PCA; Correlation | | Adesakin et al., 2020 |
| Not reported | ASTM D5072; Literature | | | Not reported | | | | Not applicable | | Mean, Min, Max | | | Separately | | | Not reported | | | Correlation coefficient | | Bello et al., 2020 |
| 10 | Literature | | | Not reported | | | | Not applicable | | Individual values | | |  | | | Not applicable | | | Not reported | | Shu’aibu et al., 2020 |
| Not reported | Not reported | | | Not reported | | | | Not applicable | | Mean, Min, Max, SD, SE | | | Mean ± SD | | | Not reported | | | PCA; ANOVA; Pearson correlation | | Ubuoh et al., 2019 |
| 30 | Not reported | | | Not reported | | | | Zero | | Individual values | | |  | | | Not reported | | | PCA; CA | | Onwuka et al., 2019 |
| 120 | APHA 1999; USEPA 2001 | | | Not reported | | | | Zero | | Mean, SD | | | Mean ± SD | | | Not reported | | | ANOVA; t-test | | Ekere et al., 2019 |
| 12 | Not reported | | | Not reported | | | | Not applicable | | Mean, Min, Max | | | Separately | | | Not applicable | | | Not reported | | Adiat et al., 2019 |
| 14 | APHA, 1998 | | | Not reported | | | | <IDL; ND | | Mean, Min, Max | | | Separately | | | Not reported | | | PCA; Pearson correlation | | Aromolaran et al., 2019 |
| 20 | Not reported | | | Not reported | | | | Not applicable | | Mean, Min, Max, SD | | | Separately | | | Not applicable | | | Not reported | | Ibrahim et al., 2019 |
| 124 | USEAPA, 2005; ASTM D1976 | | | Not reported | | | | Zero | | Med, Mean, Min, Max, SD | | | Separately | | | Shapiro-Wilk; Q-Q plots; Kolmogorov- Smirnov, ANOVA | | | PCA; CA; Pearson correlation | | Nnorom et al., 2019 |
| 20 | USEPA, 2013; APHA, 2005 | | | z-scale standardization | | | | Zero | | Individual values | | |  | | | Not reported | | | ANOVA; PCA; CA; Pearson correlation | | Omonoma et al., 2019 |
| 150 | APHA, 2005 | | | Not reported | | | | Zero | | Mean, Min, Max, SD, CV | | | Separately | | | Not reported | | | Pearson correlation | | Owoyemi et al., 2019 |
| Not reported | APHA, 1994 | | | Not reported | | | | Zero | | Individual values | | |  | | | Not reported | | | ANOVA | | Turajo et al., 2019 |
| 36 | Not reported | | | Not reported | | | | <IDL | | Mean, SD, CV | | | Separately | | | Not applicable | | | Not reported | | Daramola et al., 2019 |
| Not reported | APHA, 1998 | | | Not reported | | | | Not applicable | | Individual values | | |  | | | Not reported | | | PCA; ANOVA | | Ezemonye et al., 2019 |
| 18 | Literature | | | Not reported | | | | ND | | Mean, SD | | | Mean ± SD | | | Not reported | | | Correlation | | Ibe et al., 2019 |
| 144 | APHA, 2005 | | | Not reported | | | | Not applicable | | Mean, SD | | | Mean ± SD | | | Not reported | | | ANOVA; PCA; CA | | Adesiyan et al., 2018 |
| Not reported | Literature | | | Not reported | | | | Zero; ND | | Individual values | | |  | | | Not reported | | | ANOVA; Pearson correlation | | Titilawo et al., 2018 |
| 21 | APHA, 2005 | | | Not reported | | | | Zero | | Mean, Min, Max, SD, Q1-Q3, CV | | | Separately | | | Kurtosis, Skewness | | | Pearson correlation; PCA; CA | | P. G. C. Emenike et al., 2018 |
| 63 | APHA, 2005 | | | Not reported | | | | Not reported | | Mean, Min, Max, Q1-Q3 | | | Separately | | | Not reported | | | Pearson correlation; PCA; CA | | C. P. G. Emenike et al., 2018 |
| 5 | Not reported | | | Not reported | | | | Not applicable | | Individual values | | |  | | | Not applicable | | | Not reported | | Odipe et al., 2018 |
| Not reported | APHA, 1998 | | | Not reported | | | | Not applicable | | Mean, SD | | | Mean ± SD | | | Not reported | | | Correlation | | Bashiru et al., 2018 |
| Not reported | APHA, 2002 | | | Not reported | | | | Not applicable | | Mean, SE | | | Mean ± SE | | | Not reported | | | Correlation; ANOVA | | Ibanga et al., 2018 |
| 3 | Not reported | | | Not reported | | | | Not applicable | | Individual values | | |  | | | Not applicable | | | Not reported | | Joe-Ukairo & Oni, 2018 |
| 15 | Literature | | | Not reported | | | | ND | | Individual values | | |  | | | Not applicable | | | Not reported | | Ukah et al., 2018 |
| Not reported | APHA, 1998 | | | Not reported | | | | Not reported | | Individual values | | |  | | | Not applicable | | | Not reported | | Fakayode & Ogunjobi, 2018 |
| Not reported | APHA, 2012 | | | Not reported | | | | ND | | Individual values | | |  | | | Not reported | | | ANOVA | | Ayandiran et al., 2018 |
| Not reported | APHA, 2005 | | | Not reported | | | | Not reported | | Individual values | | |  | | | Not reported | | | ANOVA | | Ejike et al., 2017 |
| 25 | Not reported | | | Not reported | | | | Not applicable | | Mean, Min, Max, SD | | | Separately | | | Not reported | | | Correlation; FA | | A. M. Odukoya et al., 2017 |
| 75 | Not reported | | | Not reported | | | | Not applicable | | Individual values | | |  | | | Not reported | | | Bartlett’s test; Chi-Square | | Usese et al., 2017 |
| 58 | Not reported | | | Not reported | | | | Not applicable | | Individual values | | |  | | | Not reported | | | PCA; Pearson correlation; ANOVA | | Omaka et al., 2017 |
| 4 | AOAC, 2005; APHA, 2005 | | | Not reported | | | | ND | | Mean, SD | | | Mean ± SD | | | Not applicable | | | Not reported | | Sawyerr et al., 2017 |
| 20 | Not reported | | | Not reported | | | | Not applicable | | Mean, Min, Max | | | Separately | | | Not applicable | | | Not reported | | Stephen et al., 2017 |
| Not reported | APHA, 2005; DPR, 2002; Literature | | | Not reported | | | | Not applicable | | Mean, SD | | | Separately | | | Not applicable | | | Not reported | | Ifelebuegu et al., 2017 |
| 36 | Not reported | | | Not reported | | | | Not applicable | | Mean, Min, Max, SD | | | Separately | | | Not reported | | | PCA; Correlation; | | P. G. C. Emenike et al., 2017 |
| 120 | APHA, 1998 | | | Not reported | | | | Zero | | Mean, SD | | | Mean ± SD | | | Not reported | | | ANOVA; Pearson correlation | | Igbinosa & Aighewi, 2017 |
| 54 | APHA, 1998 | | | Not reported | | | | Not applicable | | Mean, Min, Max, SD | | | Separately | | | Not applicable | | | Not reported | | Tongo et al., 2017 |
| Not reported | Not reported | | | Not reported | | | | Not applicable | | Mean, SD | | | Separately | | | Not applicable | | | Not reported | | Olatunji & Ajay, 2016 |
| Not reported | APHA, 1995 | | | Not reported | | | | Not reported | | Individual values | | |  | | | Not applicable | | | Not reported | | Akintola et al., 2016 |
| 40 | Not reported | | | Not reported | | | | Not applicable | | Individual values | | |  | | | Not applicable | | | Not reported | | Osinowo, 2016 |
| 20 | APHA, 1998 | | | Not reported | | | | Zero | | Mean, Min, Max, SD, CV | | | Separately | | | Not reported | | | Pearson correlation | | (Aboyeji & Eigbokhan, 2016) |
| 12 | APHA, 2012; ASTM, D1293; ASTM, D2972 | | | Not reported | | | | Not applicable | | Mean, Min, Max, SD | | | Mean ± SD | | | Not reported | | | ANOVA; Multiple linear regression | | Omaka et al., 2016 |
| 12 | APHA, 1992, 2012; Literature | | | Not reported | | | | ND, zero | | Mean, Min, Max, SD | | | Separately | | | Not reported | | | ANOVA; correlation | | Sojobi, 2016 |
| Not reported | APHA, 1985 | | | Not reported | | | | Not reported | | Individual values | | |  | | | Not applicable | | | CA | | Ogunkunle et al., 2016 |
| 170 | APHA, 1998 | | | Not reported | | | | <MDL | | Individual values | | |  | | | Not reported | | | Pearson correlation; Duncan’s test | | Ayedun et al., 2015 |
| 50 | Not reported | | | Not reported | | | | Zero | | Mean, Min, Max, SD | | | Separately | | | Not reported | | | PCA; Pearson correlation | | Nganje et al., 2015 |
| 60 | Not reported | | | Not reported | | | | Zero | | Mean, Min, Max, SD | | | Separately | | | Not reported | | | PCA; Correlation | | Adamu et al., 2015 |
| Not reported | Not reported | | | Not reported | | | | Not applicable | | Individual values | | |  | | | Not applicable | | | Not reported | | Vincent-Akpu et al., 2015 |
| 15 | Not reported | | | Not reported | | | | Not reported | | Individual values | | |  | | | Not applicable | | | Not reported | | Okorie et al., 2015 |
| 40 | Literature | | | Not reported | | | | <IDL | | Individual values | | |  | | | Not applicable | | | Not reported | | Igwe et al., 2015 |
| Not reported | APHA, 1998; Literature | | | Not reported | | | | Not reported | | Mean, SD | | | Mean ± SD | | | Not reported | | | ANOVA; PCA | | Chia & Kwaghe, 2015 |
| Not reported | APHA, 1995; USEPA, 200.8 | | | Not reported | | | | Not applicable | | Individual values | | |  | | | Not applicable | | | Not reported | | Maxwell et al., 2015 |
| 15 | APHA, 1998 | | | Not reported | | | | Not reported | | Mean, SD | | | Mean ± SD | | | Not reported | | | Paired t-test | | Akoteyon, 2014 |
| 42 | USEPA, 2005; Literature | | | z-scale standardization | | | | Zero | | Mean, Min, Max, SD | | | Separately | | | Not reported | | | PCA; DA; CA; | | O. V. Omonona et al., 2014 |
| 72 | APHA, 2012; APHA, 1992 | | | Not reported | | | | Not applicable | | Mean, SD | | | Mean ± SD | | | Not reported | | | ANOVA | | Dahunsi et al., 2014 |
| Not reported | Not reported | | | z-scale standardization | | | | Zero | | SD, Min, Max | | | Separately | | | Kolmogorov-Smirnov test | | | CA, PCA/FA | | Ogwueleka, 2014 |
| Not reported | APHA, 1998 | | | Not reported | | | | Not applicable | | Mean, Min, Max, SE | | | Mean ± SE | | | Not reported | | | ANOVA | | Anyanwu et al., 2013 |
| 228 | APHA, 1992 | | | Not reported | | | | Not applicable | | Mean, SD | | | Mean ± SD | | | Not reported | | | Pearson correlation; ANOVA; t-test; | | Chigor et al., 2012 |
| 30 | Literature | | | Not reported | | | | Not applicable | | Individual values | | |  | | | Not applicable | | | Not reported | | Eneji et al., 2012 |
| 30 | Not reported | | | Log-transformation; z-scale standardization | | | | Zero | | Mean, SD, Min, Max | | | Separately | | | Kurtosis; Skewness | | | Not reported | | A. Mustapha & Aris, 2012 |
| 33 | Not reported | | | Not reported | | | | Not applicable | | Individual values | | |  | | | Not applicable | | | Not reported | | Zume, 2011 |
| 90 | Literature | | | Not reported | | | | Not applicable | | Mean, Min, Max, SD | | | Mean ± SD | | | Not reported | | | ANOVA | | Adediji et al., 2011 |
| Not reported | Literature | | | Not reported | | | | ND | | Mean, SE | | | Mean ± SE | | | Not reported | | | ANOVA, t-test | | Nduka & Orisakwe, 2011 |
| 25 | APHA, 1981; Literature | | | Not reported | | | | Not applicable | | Mean, SE | | | Mean ± SE | | | Not reported | | | ANOVA | | Kolawole et al., 2011 |
| 15 | Literature | | | Not reported | | | | Not applicable | | Individual values | | |  | | | Not reported | | | F-test | | O. O. Odukoya et al., 2010 |
| Not reported | APHA, 1998 | | | Not reported | | | | Zero | | Individual values | | |  | | | Not applicable | | | Not reported | | Adesalu et al., 2010 |
| 72 | APHA, 1995,1998; Literature | | | Not reported | | | | Not applicable | | Mean, SD, Min, Max, CV | | | Separately | | | Not applicable | | | Not reported | | Williams & Benson, 2010 |
| Not reported | APHA, 1995 | | | Not reported | | | | Not applicable | | Individual values | | |  | | | Not reported | | | ANOVA | | M. K. Mustapha, 2009 |
| 256 | Not reported | | | Not reported | | | | Not applicable | | Mean, SD, Min, Max, Med | | | Separately | | | Not reported | | | t-test | | Edet & Worden, 2009 |
| 6 | Literature | | | Not reported | | | | Zero | | Mean, SD, Min, Max | | | Mean ± SD | | | Not reported | | | ANOVA | | Achudume, 2009 |
| Not reported | APHA, 1998 | | | Not reported | | | | <MDL | | Mean, SD | | | Mean ± SD | | | Not reported | | | Kruskal-Wallis ANOVA; Correlation | | Ipeaiyeda & Onianwa, 2009 |
| Not reported | APHA, 1995; Literature | | | Not reported | | | | Not applicable | | Individual values | | |  | | | Not reported | | | ANOVA | | M. K. Mustapha, 2008 |
| 137 | APHA, 1995 | | | z-scale standardization | | | | Zero | | Mean, SD, Min, Max | | | Separately | | | Not reported | | | FA, PCA, CA | | Omo-Irabor et al., 2008 |
| Not reported | Literature | | | Not reported | | | | ND | | Individual values | | |  | | | Not applicable | | | Not reported | | Adesalu & Nwankwo, 2008 |
| 22 | Not reported | | | Not reported | | | | Not applicable | | Mean, SD | | | Mean ± SD | | | Not applicable | | | Not reported | | Ejechi et al., 2007 |
| 15 | APHA, 1992 | | | Not reported | | | | Not applicable | | Individual values | | |  | | | Not applicable | | | Not reported | | Rim-Rukeh et al., 2007 |
| 12 | Not reported | | | Not reported | | | | Not applicable | | Mean, SE | | | Separately | | | Not reported | | | Pearson correlation; ANOVA | | I. M. Adekunle et al., 2007 |
| 234 | Not reported | | | Not reported | | | | Zero | | Mean, SD | | | Mean ± SD | | | Not reported | | | ANOVA | | Jaji et al., 2007 |
| Not reported | APHA, 1985 | | | Not reported | | | | Not applicable | | Mean, SE | | | Mean ± SE | | | Not reported | | | ANOVA | | Arimoro et al., 2007a |
| Not reported | APHA, 1985 | | | Not reported | | | | Not applicable | | Mean, SE | | | Mean ± SE | | | Not reported | | | Pearson correlation; ANOVA | | Arimoro et al., 2007b |
| 41 | Not reported | | | Not reported | | | | Not applicable | | Mean, Min, Max, SE | | | Separately | | | Not reported | | | Not reported | | Orisakwe et al., 2006 |
| Not reported | Not reported | | | Not reported | | | | <IDL | | Mean, SE | | | Mean ± SE | | | Not applicable | | | Not reported | | Igwilo et al., 2006 |
| Not reported | APHA, 1992 | | | Not reported | | | | <IDL | | Mean, SD | | | Mean ± SD | | | Not reported | | | ANOVA | | Uzoukwu et al., 2004 |
| Not reported | Literature | | | Not reported | | | | Not applicable | | Individual values | | |  | | | Not applicable | | | Not reported | | Okafor & Ogbonna, 2003 |
| 22 | Literature | | | Not reported | | | | Zero | | Med, Min, Max | | | Separately | | | Not applicable | | | Not reported | | Aremu et al., 2002 |
| 51 | APHA, 1985 | | | Not reported | | | | Not applicable | | Mean, SD | | | Mean ± SD | | | Not reported | | | F-test; ANOVA; Mann-Whitney; Kruskal-Wallis | | Ikem et al., 2002 |
| Not reported | APHA, 1989; Literature | | | Not reported | | | | ND | | Mean, Min, Max | | | Separately | | | Not reported | | | t-test | | Olajire & Imeokparia, 2001 |
| **Trace organics** | | |  | | |  | | |  | | |  | | |  | | |  | |  |  |
| 37 | Literature | | | Not reported | | | | <MDL | | Median, Min, Max | | | Separately | | | Kolmogorov-Smirnov test; Q-Q plot | | | t-test; ANOVA | | (Ebele et al., 2020) |
| Not reported | Literature | | | Not reported | | | | Not applicable | | Individual values | | |  | | | Shapiro-Wilk test | | | ANOVA; Chi-square | | (Ogunbanwo et al., 2020) |
| 4 | Not reported | | | Not reported | | | | <LoQ | | Individual values | | |  | | | Not applicable | | | Not reported | | (Aganbi et al., 2019) |
| 216 | USEPA, 2007 | | | Not reported | | | | Not applicable | | Mean, SD | | | Mean ± SD | | | Shapiro-Wilk test | | | Not reported | | (Chukwuka et al., 2019) |
| Not reported | Literature | | | Not reported | | | | Not applicable | | Individual values | | |  | | | Not reported | | | t-test | | (Lan et al., 2019) |
| 144 | Not reported | | | Not reported | | | | ND | | Mean, SD | | | Mean ± SD | | | Not reported | | | Not reported | | (Ogbeide et al., 2019) |
| 5 | Literature | | | Not reported | | | | Zero | | Individual values | | |  | | | Not reported | | | PCA; ANOVA; Pearson correlation | | (Onyekwelu & Aghamelu, 2019) |
| Not reported | Literature | | | Not reported | | | | Not applicable | | Individual values | | |  | | | Not reported | | | ANOVA | | (Sogbanmu et al., 2019) |
| Not reported | Literature | | | Not reported | | | | Not applicable | | Mean, SD | | | Mean ± SD | | | Not reported | | | Not reported | | (Ololade et al., 2018) |
| 72 | Not reported | | | Not reported | | | | BDL | | Individual values | | |  | | | Not reported | | | ANOVA; PCA; CA | | (A. S. Adekunle et al., 2017) |
| 6 | Literature | | | Not reported | | | | <DL | | Mean, SD | | | Separately | | | Not reported | | | Not reported | | (Ifelebuegu et al., 2017) |
| 54 | USEPA 600 | | | Not reported | | | | ND | | Mean, SD, Min, Max | | | Separately | | | Not reported | | | PCA; ANOVA; Correlation | | (Tongo et al., 2017) |
| 5 | Not reported | | | Not reported | | | | BDL | | Individual values | | |  | | | Not reported | | | Not reported | | (Inam et al., 2015) |
| 33 | Not reported | | | Not reported | | | | Zero | | Individual values | | |  | | | Not reported | | | Not reported | | (Zume, 2011) |
| 15 | Literature | | | Not reported | | | | ND | | Mean, SD, Min, Max | | | Separately | | | Not reported | | | Not reported | | (O. O. Odukoya et al., 2010) |
| 137 | APHA, 1995 | | | *z*-scale standardization | | | | Zero | | Mean, SD, Min, Max | | | Separately | | | Not reported | | | PCA; CA; Correlation | | (Omo-Irabor et al., 2008) |
|  |  | | |  | | | |  | |  | | |  | | |  | | |  | |  |
| **Bacteriological parameters** | | |  | | |  | | |  | | |  | | |  | | |  | |  |  |
| Not reported | APHA, 2001 | | | Not reported | | | |  | | Mean, SE, Min, Max | | | Mean ± SE | | |  | | | ANOVA; T-test; Pearson correlation; PCA | | (Adesakin et al., 2020) |
| 54 | Literature | | | Not reported | | | |  | | Mean, SD, Min, Max | | | Separately | | |  | | | Paired T-test; ANOVA; Pearson correlation | | (Alum & Okoye, 2020) |
| 72 | USEPA, 2009; Literature | | | z-scale transformation | | | |  | | Mean, SD, V | | | Separately | | |  | | | T-test; Pearson’s correlation; chi-square | | (Ocheli et al., 2020) |
| 21 | APHA, 2012; Literature | | | Not reported | | | |  | | Mean, SE, | | | Mean ± SE | | |  | | | ANOVA; DMRT | | (Opafola et al., 2020) |
| 24 | Literature | | | Not reported | | | |  | | Mean, SD | | | Mean ± SD | | |  | | | PCA | | (Imarhiagbe & Osarenotor, 2020) |
| 14 | Not reported | | | Not applicable | | | |  | | Mean, Min, Max | | | Separately | | |  | | | Descriptive | | (Aromolaran et al., 2019) |
| 15 | APHA, 1989; Literature | | | Not applicable | | | |  | | Individual values | | |  | | |  | | | Descriptive | | (Ukah et al., 2018) |
| Not reported | Not reported | | | Not applicable | | | |  | | Individual values | | |  | | |  | | | Descriptive | | (Fakayode & Ogunjobi, 2018) |
| 10 | APHA, 1998 | | | Not applicable | | | |  | | Individual values | | |  | | |  | | | Descriptive | | (Aboh et al., 2015b) |
| 40 | Literature | | | Not applicable | | | |  | | Individual values | | |  | | |  | | | Descriptive | | (Igwe et al., 2015) |
| Not reported | Not reported | | | z-scale transformation | | | |  | | SD, Min, Max | | | Separately | | |  | | | PCA | | (Ogwueleka, 2014) |
| 72 | APHA, 2012 | | | Not applicable | | | |  | | Mean, SD | | | Mean ± SD | | |  | | | Descriptive | | (Dahunsi et al., 2014) |
| 53 | Not reported | | | Not applicable | | | |  | | Individual values | | |  | | |  | | | Descriptive | | (Igbenegbu & Lamikanra, 2014) |
| Not reported | APHA, 1992 | | | Not reported | | | |  | | Mean, SD | | | Mean ± SD | | |  | | | Pearson’s correlation; ANOVA; DMRT | | (Chigor et al., 2012) |
| Not reported | Not reported | | | Log-transformed;  z-scale standardized | | | |  | | Mean, SD, Min, Max | | | Separately | | |  | | | HACA; PCA; DA | | (A. Mustapha & Aris, 2012) |
| Not reported | Not reported | | | Not reported | | | |  | | Individual values | | |  | | |  | | | Chi-sqaure | | (Akinyemi et al., 2010) |
| 25 | Literature | | | Not applicable | | | |  | | Mean, SE | | | Mean ± SE | | |  | | | Descriptive | | (Kolawole et al., 2011) |
| Not reported | Not reported | | | Not applicable | | | |  | | Individual values | | |  | | |  | | | Descriptive | | (Olaoye & Onilude, 2009) |
| 12 | Not reported | | | Not reported | | | |  | | Mean, SD | | | Mean ± SD | | |  | | | ANOVA; Pearson’s correlation | | (I. M. Adekunle et al., 2007) |
| 234 | Not reported | | | Not reported | | | |  | | Mean, SD | | | Mean ± SD | | |  | | | ANOVA; DMRT | | (Jaji et al., 2007) |

Min= Minimum; Max= Maximum; SD= Standard deviation; SE= Standard error; V= Variance; CV= Coefficient of variation; ANOVA= Analysis of variance; *Not applicable= sample values are all greater than zero

Table S4. Comparative summary of the data collection, analysis and reporting approaches for physicochemical parameters in other countries

| Standard protocol for data collection | Standardization and transformation | Reporting style for rounded zeros | Normality test | Chosen statistics | Country | Reference |
| --- | --- | --- | --- | --- | --- | --- |
| Not reported | Not reported | No zeros | Boxplots | PCA; Correlation | Sri Lanka | (Koliyabandara et al., 2020) |
| APHA, 1999 | z- scale standardization | <DL | Not reported | PCA | Angola | (Paca et al., 2019) |
| APHA, 1998 | Not reported | No zeros | Skewness & Kurtosis | PCA; Pearson correlation | Bangladesh | (Howladar et al., 2021) |
| APHA, 1999; Literature | z- scale standardization | No zeros | Not reported | PCA; ANOVA; t-test | Ghana | (Miyittah et al., 2020) |
| Not reported | Not reported | <DL | Not reported | PCA; Correlation | China | (Chai et al., 2021) |
| Not reported | Log-transformed | No zeros | Not reported | CA; Pearson correlation | China | (Wu et al., 2021) |
| Not reported | Not reported | No zeros | Boxplots | Pearson correlation | China | (Y. Liu et al., 2021) |
| APHA 1992 | z- scale standardization | No zeros | Not reported | Spearman correlation | India | (Singh et al., 2004) |
| Not reported | Not reported | ND | Not reported | PCA; Pearson correlation | China | (Meng et al., 2016) |
| APHA 3500B; 2340B | z- scale standardization | No zeros | Not reported | FA; Correlation | Taiwan | (C. W. Liu et al., 2003) |
| CSEPB, 2002 | Not reported | Zero; ND | Not reported | FA/PCA; ANOVA; CA | China | (Li et al., 2011) |
| China EPA, 2009 | z- scale standardization | No zeros | Kolmogorov-Smirnov test | FA/PCA; ANOVA; CA; Correlation | China | (Wang et al., 2017) |
| Not reported | z- scale standardization | Zero | Shapiro-Wick test | CA; FA/PCA; Spearman correlation | Nepal | (Chapagain et al., 2010) |

**References**

Aboh, E. A., Giwa, F. J., & Giwa, A. (2015a). Microbiological assessment of well waters in Samaru, Zaria, Kaduna, State, Nigeria. 14(1). 32-38. https://doi.org/10.4103/1596-3519.148732

Aboh, E. A., Giwa, F. J., & Giwa, A. (2015b). Microbiological assessment of well waters in Samaru, Zaria, Kaduna, State, Nigeria. *Annals of African Medicine*, *14*(1), 32–38. https://doi.org/10.4103/1596-3519.148732

Aboyeji, O. S., & Eigbokhan, S. F. (2016). Evaluations of groundwater contamination by leachates around Olusosun open dumpsite in Lagos metropolis, southwest Nigeria. *Journal of Environmental Management*, *183*, 333–341. https://doi.org/10.1016/j.jenvman.2016.09.002

Achudume, A. C. (2009). The effect of petrochemical effluent on the water quality of ubeji creek in niger delta of nigeria. *Bulletin of Environmental Contamination and Toxicology*, *83*(3), 410–415. https://doi.org/10.1007/s00128-009-9736-2

Adamu, C. I., Nganje, T. N., & Edet, A. (2015). Heavy metal contamination and health risk assessment associated with abandoned barite mines in Cross River State, southeastern Nigeria. *Environmental Nanotechnology, Monitoring and Management*, *3*, 10–21. https://doi.org/10.1016/j.enmm.2014.11.001

Adediji, A., Adewumi, J. A., & Ologunorisa, T. E. (2011). Effects of irrigation on the physico-chemical quality of water in irrigated areas: The Upper Osin Catchment, Kwara State, Nigeria. *Progress in Physical Geography*, *35*(6), 707–719. https://doi.org/10.1177/0309133311407655

Adekunle, A. S., Oyekunle, J. A. O., Ojo, O. S., Maxakato, N. W., Olutona, G. O., & Obisesan, O. R. (2017). Determination of polycyclic aromatic hydrocarbon levels of groundwater in Ife north local government area of Osun state, Nigeria. *Toxicology Reports*, *4*, 39–48. https://doi.org/10.1016/j.toxrep.2016.10.002

Adekunle, I. M., Adetunji, M. T., Gbadebo, A. M., & Banjoko, O. B. (2007). Assessment of groundwater quality in a typical rural settlement in southwest Nigeria. *International Journal of Environmental Research and Public Health*, *4*(4), 307–318. https://doi.org/10.3390/ijerph200704040007

Adesakin, T. A., Oyewale, A. T., Bayero, U., Mohammed, A. N., Aduwo, I. A., Ahmed, P. Z., Abubakar, N. D., & Barje, I. B. (2020). Assessment of bacteriological quality and physico-chemical parameters of domestic water sources in Samaru community, Zaria, Northwest Nigeria. *Heliyon*, *6*(8), e04773. https://doi.org/10.1016/j.heliyon.2020.e04773

Adesalu, T., Bagbe, M., & Keyede, D. (2010). Hydrochemistry and phytoplankton composition of two tidal creeks in south-western Nigeria. *Revista de Biologia Tropical*, *58*(3), 827–840. https://doi.org/10.15517/rbt.v58i2.5249

Adesalu, T., & Nwankwo, D. I. (2008). Effect of Water Quality Indicies on Phytoplankton of a Sluggish Tidal Creeks in Lagos, Nigeria. *Pakistan Journal of Biological Sciences*, *11*(6), 836–844. https://scialert.net/fulltext/?doi=pjbs.2008.836.844&org=11

Adesiyan, I. M., Bisi-Johnson, M., Aladesanmi, O. T., Okoh, A. I., & Ogunfowokan, A. O. (2018). Concentrations and human health risk of heavy metals in rivers in Southwest Nigeria. *Journal of Health and Pollution*, *8*(19). https://doi.org/10.5696/2156-9614-8.19.180907

Adiat, K. A. N., Adegoroye, A. A., Adebiyi, A. D., Akeredolu, B. E., & Akinlalu, A. A. (2019). Comparative assessment of aquifer susceptibilities to contaminant from dumpsites in different geological locations. *Heliyon*, *5*(5), e01499. https://doi.org/10.1016/j.heliyon.2019.e01499

Adiat, K. A. N., Akeredolu, B. E., Akinlalu, A. A., & Olayanju, G. M. (2020). Application of logistic regression analysis in prediction of groundwater vulnerability in gold mining environment: a case of Ilesa gold mining area, southwestern, Nigeria. *Environmental Monitoring and Assessment*, *192*(9). https://doi.org/10.1007/s10661-020-08532-7

Afonne, O. J., Chukwuka, J. U., & Ifediba, E. C. (2020). Evaluation of drinking water quality using heavy metal pollution indexing models in an agrarian, non-industrialised area of South-East Nigeria. *Journal of Environmental Science and Health - Part A Toxic/Hazardous Substances and Environmental Engineering*, *0*(0), 1–9. https://doi.org/10.1080/10934529.2020.1796402

Aganbi, E., Iwegbue, C. M. A., & Martincigh, B. S. (2019). Concentrations and risks of polychlorinated biphenyls (PCBs) in transformer oils and the environment of a power plant in the Niger Delta, Nigeria. *Toxicology Reports*, *6*(August), 933–939. https://doi.org/10.1016/j.toxrep.2019.08.008

Akintola, O. A., Sangodoyin, A. Y., & Agunbiade, F. O. (2016). Fuzzy Logic Modelling of the Effects of Pollution on Domestic Roof-Harvested Rainwater Quality in Residential and Industrial Environments. *Archives of Environmental Contamination and Toxicology*, *71*(1), 113–121. https://doi.org/10.1007/s00244-016-0278-4

Akinyemi, K. O., Iwalokun, B. A., Foli, F., Oshodi, K., & Coker, A. O. (2010). Prevalence of multiple drug resistance and screening of enterotoxin ( stn ) gene in Salmonella enterica serovars from water sources in Lagos , Nigeria. *Public Health*, *125*(2), 65–71. https://doi.org/10.1016/j.puhe.2010.11.010

Akoteyon, I. S. (2014). Seasonal Variations of Shallow Well Water Quality in Amuwo-Odofin and Ojo LGA’s of Lagos, Nigeria. *Environmental Research, Engineering and Management*, *68*(2), 5–14. https://doi.org/10.5755/j01.erem.68.2.5642

Alum, O. L., & Okoye, C. O. B. (2020). Pollution status of major rivers in an agricultural belt in Eastern Nigeria. *Environmental Monitoring and Assessment*, *192*(6). https://doi.org/10.1007/s10661-020-08366-3

Anyanwu, E. D., Ikomi, R. B., & Arimoro, F. O. (2013). Water quality and zooplankton of the Ogba River, Benin City, Nigeria. *African Journal of Aquatic Science*, *38*(2), 193–199. https://doi.org/10.2989/16085914.2013.784697

Aremu, D. A., Olawuyi, J. F., Meshitsuka, S., Sridhar, M. K., & Oluwande, P. A. (2002). Heavy metal analysis of groundwater from Warri, Nigeria. *International Journal of Environmental Health Research*, *12*(3), 261–267. https://doi.org/10.1080/0960312021000001014

Arimoro, F. O., Ikomi, R. B., & Iwegbue, C. M. A. (2007a). Ecology and Abundance of Oligochaetes as Indicators of Organic Pollution in an Urban Stream in Southern Nigeria. *Pakistan Journal of Biological Sciences*, *10*(3), 446–453. https://scialert.net/fulltext/?doi=pjbs.2007.446.453&org=11

Arimoro, F. O., Ikomi, R. B., & Iwegbue, C. M. A. (2007b). Water quality changes in relation to Diptera community patterns and diversity measured at an organic effluent impacted stream in the Niger Delta, Nigeria. *Ecological Indicators*, *7*(3), 541–552. https://doi.org/10.1016/j.ecolind.2006.06.002

Aromolaran, O., Fagade, O. E., Aromolaran, O. K., Faleye, E. T., & Faerber, H. (2019). Assessment of groundwater pollution near Aba-Eku municipal solid waste dumpsite. *Environmental Monitoring and Assessment*, *191*(12), 1–25. https://doi.org/10.1007/s10661-019-7886-1

Ayandiran, T. A., Fawole, O. O., & Dahunsi, S. O. (2018). Water quality assessment of bitumen polluted Oluwa River, South-Western Nigeria. *Water Resources and Industry*, *19*(December 2017), 13–24. https://doi.org/10.1016/j.wri.2017.12.002

Ayedun, H., Gbadebo, A. M., Idowu, O. A., & Arowolo, T. A. (2015). Toxic elements in groundwater of Lagos and Ogun States, Southwest, Nigeria and their human health risk assessment. *Environmental Monitoring and Assessment*, *187*(6). https://doi.org/10.1007/s10661-015-4319-7

Bamigboye, C. O., Amao, J. A., Ayodele, T. A., Adebayo, A. S., Ogunleke, J. D., Abass, T. B., Oyedare, T. A., Adetutu, T. J., Adeeyo, A. O., & Oyedemi, A. A. (2020). An appraisal of the drinking water quality of groundwater sources in Ogbomoso, Oyo state, Nigeria. *Groundwater for Sustainable Development*, *11*, 100453. https://doi.org/10.1016/j.gsd.2020.100453

Bello, S., Nasiru, R., Garba, N. N., & Adeyemo, D. J. (2020). Annual effective dose associated with radon, gross alpha and gross beta radioactivity in drinking water from gold mining areas of Shanono and Bagwai, Kano state, Nigeria. *Microchemical Journal*, *154*(October 2019), 104551. https://doi.org/10.1016/j.microc.2019.104551

Beshiru, A., Okareh, O. T., Chigor, V. N., & Igbinosa, E. O. (2018). Assessment of water quality of rivers that serve as water sources for drinking and domestic functions in rural and pre-urban communities in Edo North, Nigeria. *Environmental Monitoring and Assessment*, *190*(7). https://doi.org/10.1007/s10661-018-6771-7

Chai, N., Yi, X., Xiao, J., Liu, T., Liu, Y., Deng, L., & Jin, Z. (2021). Spatiotemporal variations, sources, water quality and health risk assessment of trace elements in the Fen River. *Science of the Total Environment*, *757*, 143882. https://doi.org/10.1016/j.scitotenv.2020.143882

Chapagain, S. K., Pandey, V. P., Shrestha, S., Nakamura, T., & Kazama, F. (2010). Assessment of deep groundwater quality in kathmandu valley using multivariate statistical techniques. *Water, Air, and Soil Pollution*, *210*(1–4), 277–288. https://doi.org/10.1007/s11270-009-0249-8

Chia, M. A., & Kwaghe, M. J. (2015). Microcystins contamination of surface water supply sources in Zaria-Nigeria. *Environmental Monitoring and Assessment*, *187*(10). https://doi.org/10.1007/s10661-015-4829-3

Chigor, V. N., Umoh, V. J., Okuofu, C. A., Ameh, J. B., Igbinosa, E. O., & Okoh, A. I. (2012). Water quality assessment: Surface water sources used for drinking and irrigation in Zaria, Nigeria are a public health hazard. *Environmental Monitoring and Assessment*, *184*(5), 3389–3400. https://doi.org/10.1007/s10661-011-2396-9

Chukwuka, A., Ogbeide, O., & Uhunamure, G. (2019). Gonad pathology and intersex severity in pelagic (Tilapia zilli) and benthic (Neochanna diversus and Clarias gariepinus) species from a pesticide-impacted agrarian catchment, south-south Nigeria. *Chemosphere*, *225*, 535–547. https://doi.org/10.1016/j.chemosphere.2019.03.073

Dahunsi, S. O., Owamah, H. I., Ayandiran, T. A., & Oranusi, S. U. (2014). Drinking Water Quality and Public Health of Selected Towns in South Western Nigeria. *Water Quality, Exposure and Health*, *6*(3), 143–153. https://doi.org/10.1007/s12403-014-0118-6

Daramola, J., M. Ekhwan, T., Adepehin, E. J., Mokhtar, J., Lam, K. C., & Er, A. C. (2019). Seasonal quality variation and environmental risks associated with the consumption of surface water: implication from the Landzun Stream, Bida Nigeria. *Heliyon*, *5*(7), e02121. https://doi.org/10.1016/j.heliyon.2019.e02121

Ebele, A. J., Oluseyi, T., Drage, D. S., Harrad, S., & Abou-Elwafa Abdallah, M. (2020). Occurrence, seasonal variation and human exposure to pharmaceuticals and personal care products in surface water, groundwater and drinking water in Lagos State, Nigeria. *Emerging Contaminants*, *6*, 124–132. https://doi.org/10.1016/j.emcon.2020.02.004

Edet, A., & Worden, R. H. (2009). Monitoring of the physical parameters and evaluation of the chemical composition of river and groundwater in Calabar (Southeastern Nigeria). *Environmental Monitoring and Assessment*, *157*(1–4), 243–258. https://doi.org/10.1007/s10661-008-0532-y

Egbueri, J. C. (2020). Groundwater quality assessment using pollution index of groundwater (PIG), ecological risk index (ERI) and hierarchical cluster analysis (HCA): A case study. *Groundwater for Sustainable Development*, *10*(July 2019), 100292. https://doi.org/10.1016/j.gsd.2019.100292

Egbueri, J. C., Ezugwu, C. K., Ameh, P. D., Unigwe, C. O., & Ayejoto, D. A. (2020). Appraising drinking water quality in Ikem rural area (Nigeria) based on chemometrics and multiple indexical methods. *Environmental Monitoring and Assessment*, *192*(5). https://doi.org/10.1007/s10661-020-08277-3

Ejechi, B. O., Olobaniyi, S. B., Ogban, F. E., & Ugbe, F. C. (2007). Physical and sanitary quality of hand-dug well water from oil-producing area of Nigeria. *Environmental Monitoring and Assessment*, *128*(1–3), 495–501. https://doi.org/10.1007/s10661-006-9343-1

Ejike, C. E. C. C., Eferibe, C. O., & Okonkwo, F. O. (2017). Concentrations of some heavy metals in underground water samples from a Nigerian crude oil producing community. *Environmental Science and Pollution Research*, *24*(9), 8436–8442. https://doi.org/10.1007/s11356-017-8524-5

Ekere, N. R., Agbazue, V. E., Ngang, B. U., & Ihedioha, J. N. (2019). Hydrochemistry and Water Quality Index of groundwater resources in Enugu north district, Enugu, Nigeria. *Environmental Monitoring and Assessment*, *191*(3). https://doi.org/10.1007/s10661-019-7271-0

Emenike, C. P. G., Tenebe, I. T., & Jarvis, P. (2018). Fluoride contamination in groundwater sources in Southwestern Nigeria: Assessment using multivariate statistical approach and human health risk. *Ecotoxicology and Environmental Safety*, *156*(January), 391–402. https://doi.org/10.1016/j.ecoenv.2018.03.022

Emenike, P. G. C., Neris, J. B., Tenebe, I. T., Nnaji, C. C., & Jarvis, P. (2020). Estimation of some trace metal pollutants in River Atuwara southwestern Nigeria and spatio-temporal human health risks assessment. *Chemosphere*, *239*, 124770. https://doi.org/10.1016/j.chemosphere.2019.124770

Emenike, P. G. C., Nnaji, C. C., & Tenebe, I. T. (2018). Assessment of geospatial and hydrochemical interactions of groundwater quality, southwestern Nigeria. *Environmental Monitoring and Assessment*, *190*(7). https://doi.org/10.1007/s10661-018-6799-8

Emenike, P. G. C., Tenebe, T. I., Omeje, M., & Osinubi, D. S. (2017). Health risk assessment of heavy metal variability in sachet water sold in Ado-Odo Ota, South-Western Nigeria. *Environmental Monitoring and Assessment*, *189*(9). https://doi.org/10.1007/s10661-017-6180-3

Eneji, I. S., Sha’Ato, R., & Annune, P. A. (2012). An assessment of heavy metals loading in River Benue in the Makurdi Metropolitan Area in Central Nigeria. *Environmental Monitoring and Assessment*, *184*(1), 201–207. https://doi.org/10.1007/s10661-011-1959-0

Ewuzie, U., Nnorom, I. C., & Eze, S. . O. (2020). Lithium in drinking water sources in rural and urban communities in Southeastern Nigeria. *Chemosphere*, *245*, 125593. https://doi.org/10.1016/j.chemosphere.2019.125593

Eyankware, M. O., Aleke, C. G., Selemo, A. O. I., & Nnabo, P. N. (2020). Hydrogeochemical studies and suitability assessment of groundwater quality for irrigation at Warri and environs., Niger delta basin, Nigeria. *Groundwater for Sustainable Development*, *10*(October 2019), 100293. https://doi.org/10.1016/j.gsd.2019.100293

Ezemonye, L. I., Adebayo, P. O., Enuneku, A. A., Tongo, I., & Ogbomida, E. (2019). Potential health risk consequences of heavy metal concentrations in surface water, shrimp (Macrobrachium macrobrachion) and fish (Brycinus longipinnis) from Benin River, Nigeria. *Toxicology Reports*, *6*(January 2018), 1–9. https://doi.org/10.1016/j.toxrep.2018.11.010

Fakayode, I. B., & Ogunjobi, A. A. (2018). Quality assessment and prevalence of antibiotic resistant bacteria in government approved mini-water schemes in Southwest, Nigeria. *International Biodeterioration and Biodegradation*, *133*(July), 151–158. https://doi.org/10.1016/j.ibiod.2018.07.004

Gbadebo, A. M. (2020). Assessment of quality and health risk of peri-urban groundwater supply from selected areas of Abeokuta, Ogun State, Southwestern Nigeria. *Environmental Geochemistry and Health*, *5*. https://doi.org/10.1007/s10653-020-00746-5

Howladar, M. F., Chakma, E., Jahan Koley, N., Islam, S., Numanbakth, M. A. Al, Ahmed, Z., Chowdhury, T. R., & Akter, S. (2021). The water quality and pollution sources assessment of Surma river, Bangladesh using, hydrochemical, multivariate statistical and water quality index methods. *Groundwater for Sustainable Development*, *12*, 100523. https://doi.org/10.1016/j.gsd.2020.100523

Ibanga, L. B., Nkwoji, J. A., Usese, A. I., Onyema, I. C., & Chukwu, L. O. (2019). Hydrochemistry and heavy metals concentrations in sediment of Woji creek and Bonny estuary, Niger Delta, Nigeria. *Regional Studies in Marine Science*, *25*. https://doi.org/10.1016/j.rsma.2018.10.004

Ibe, F. C., Opara, A. I., Ibe, B. O., & Amaobi, C. E. (2019). Application of assessment models for pollution and health risk from effluent discharge into a tropical stream: case study of Inyishi River, Southeastern Nigeria. *Environmental Monitoring and Assessment*, *191*(12). https://doi.org/10.1007/s10661-019-7936-8

Ibrahim, K. O., Gomo, M., & Oke, S. A. (2019). Groundwater quality assessment of shallow aquifer hand dug wells in rural localities of Ilorin northcentral Nigeria: Implications for domestic and irrigation uses. *Groundwater for Sustainable Development*, *9*(July 2018), 100226. https://doi.org/10.1016/j.gsd.2019.100226

Ifelebuegu, A. O., Ukpebor, J. E., Ahukannah, A. U., Nnadi, E. O., & Theophilus, S. C. (2017). Environmental effects of crude oil spill on the physicochemical and hydrobiological characteristics of the Nun River, Niger Delta. *Environmental Monitoring and Assessment*, *189*(4). https://doi.org/10.1007/s10661-017-5882-x

Igbenegbu, O. A., & Lamikanra, A. (2014). The bacteriological quality of different brands of bottled water available to consumers in Ile-Ife ,. *BMC Research Notes*, *7*(1), 859. http://creativecommons.org/licenses/by/2.0

Igbinosa, I. H., & Aighewi, I. T. (2017). Quality assessment and public health status of harvested rainwater in a peri-urban community in Edo State of Nigeria. *Environmental Monitoring and Assessment*, *189*(8). https://doi.org/10.1007/s10661-017-6122-0

Igwe, O., Adepehin, E. J., & Adepehin, J. O. (2015). Integrated geochemical and microbiological approach to water quality assessment: case study of the Enyigba metallogenic province, South-eastern Nigeria. *Environmental Earth Sciences*, *74*(4), 3251–3262. https://doi.org/10.1007/s12665-015-4363-1

Igwilo, I. O., Afonne, O. J., Maduabuchi, U. J. M., & Orisakwe, O. E. (2006). Toxicological study of the Anam River in Otuocha, Anambra State, Nigeria. *Archives of Environmental and Occupational Health*, *61*(5), 205–208. https://doi.org/10.3200/AEOH.61.5.205-208

Ihunwo, O. C., Dibofori-Orji, A. N., Olowo, C., & Ibezim-Ezeani, M. U. (2020). Distribution and risk assessment of some heavy metals in surface water, sediment and grey mullet (Mugil cephalus) from contaminated creek in Woji, southern Nigeria. *Marine Pollution Bulletin*, *154*(February), 111042. https://doi.org/10.1016/j.marpolbul.2020.111042

Ikem, A., Osibanjo, O., Sridhar, M. K. C., & Sobande, A. (2002). Evaluation of groundwater quality characteristics near two waste sites in Ibadan and Lagos, Nigeria. *Water, Air, and Soil Pollution*, *140*(1–4), 307–333. https://doi.org/10.1023/A:1020165403531

Imarhiagbe, E. E., & Osarenotor, O. (2020). Health risk and quality assessment of non-roof-harvested rainwater from an oil-producing community in Nigeria. *Environmental Monitoring and Assessment*, *192*(3). https://doi.org/10.1007/s10661-020-8102-z

Inam, E., Offiong, N. A., Kang, S., Yang, P., & Essien, J. (2015). Assessment of the Occurrence and Risks of Emerging Organic Pollutants (EOPs) in Ikpa River Basin Freshwater Ecosystem, Niger Delta-Nigeria. *Bulletin of Environmental Contamination and Toxicology*, *95*(5), 624–631. https://doi.org/10.1007/s00128-015-1639-9

Ipeaiyeda, A. R., & Onianwa, P. C. (2009). Impact assessment of brewery effluent on water quality in Majawe, Ibadan, Southwestern Nigeria. *Chemistry and Ecology*, *25*(3), 189–204. https://doi.org/10.1080/02757540902970314

Jagaba, A. H., Kutty, S. R. M., Hayder, G., Baloo, L., Abubakar, S., Ghaleb, A. A. S., Lawal, I. M., Noor, A., Umaru, I., & Almahbashi, N. M. Y. (2020). Water quality hazard assessment for hand dug wells in Rafin Zurfi, Bauchi State, Nigeria. *Ain Shams Engineering Journal*, *xxxx*. https://doi.org/10.1016/j.asej.2020.02.004

Jaji, M. O., Bamgbose, O., Odukoya, O. O., & Arowolo, T. A. (2007). Water quality assessment of Ogun river, South West Nigeria. *Environmental Monitoring and Assessment*, *133*(1–3), 473–482. https://doi.org/10.1007/s10661-006-9602-1

Joe-Ukairo, A., & Oni, A. G. (2018). Geophysical and hydro-chemical investigations of Oke Asunle Dumpsite in Ile-Ife, Southwestern Nigeria for Subsoil and Surface Water Pollution. *Journal of Health and Pollution*, *8*(20), 1–9. https://doi.org/10.5696/2156-9614-8.20.181209

Kolawole, O. M., Ajayi, K. T., Olayemi, A. B., & Okoh, A. I. (2011). Assessment of water quality in Asa River (Nigeria) and its indigenous Clarias gariepinus fish. *International Journal of Environmental Research and Public Health*, *8*(11), 4332–4352. https://doi.org/10.3390/ijerph8114332

Koliyabandara, S. M. P. A., Asitha, T. C., Sudantha, L., & Siriwardana, C. (2020). Assessment of the impact of an open dumpsite on the surface water quality deterioration in Karadiyana, Sri Lanka. *Environmental Nanotechnology, Monitoring and Management*, *14*(September), 100371. https://doi.org/10.1016/j.enmm.2020.100371

Lan, S. M., Amaeze, N. H., Obanya, H. E., & Okoroafor, C. U. (2019). Occurrence of selected pharmaceuticals in industrial wastewater, receiving waters and fish. *African Journal of Aquatic Science*, *44*(4), 401–408. https://doi.org/10.2989/16085914.2019.1680339

Li, S., Li, J., & Zhang, Q. (2011). Water quality assessment in the rivers along the water conveyance system of the Middle Route of the South to North Water Transfer Project (China) using multivariate statistical techniques and receptor modeling. *Journal of Hazardous Materials*, *195*, 306–317. https://doi.org/10.1016/j.jhazmat.2011.08.043

Liu, C. W., Lin, K. H., & Kuo, Y. M. (2003). Application of factor analysis in the assessment of groundwater quality in a blackfoot disease area in Taiwan. *Science of the Total Environment*, *313*(1–3), 77–89. https://doi.org/10.1016/S0048-9697(02)00683-6

Liu, Y., Hu, Y., Hu, Y., Gao, Y., & Liu, Z. (2021). Water quality characteristics and assessment of Yongding New River by improved comprehensive water quality identification index based on game theory. *Journal of Environmental Sciences (China)*, *104*, 40–52. https://doi.org/10.1016/j.jes.2020.10.021

Maxwell, O., Wagiran, H., Lee, S. K., Embong, Z., & Ugwuoke, P. E. (2015). Radioactivity level and toxic elemental concentration in groundwater at Dei-Dei and Kubwa areas of Abuja, north-central Nigeria. *Radiation Physics and Chemistry*, *107*, 23–30. https://doi.org/10.1016/j.radphyschem.2014.09.003

Meng, Q., Zhang, J., Zhang, Z., & Wu, T. (2016). Geochemistry of dissolved trace elements and heavy metals in the Dan River Drainage (China): distribution, sources, and water quality assessment. *Environmental Science and Pollution Research*, *23*(8), 8091–8103. https://doi.org/10.1007/s11356-016-6074-x

Miyittah, M. K., Tulashie, S. K., Tsyawo, F. W., Sarfo, J. K., & Darko, A. A. (2020). Assessment of surface water quality status of the Aby Lagoon System in the Western Region of Ghana. *Heliyon*, *6*(7). https://doi.org/10.1016/j.heliyon.2020.e04466

Mustapha, A., & Aris, A. Z. (2012). Spatial aspects of surface water quality in the Jakara Basin, Nigeria using chemometric analysis. *Journal of Environmental Science and Health - Part A Toxic/Hazardous Substances and Environmental Engineering*, *47*(10), 1455–1465. https://doi.org/10.1080/10934529.2012.673305

Mustapha, M. K. (2008). Assessment of the water quality of oyun reservoir, Offa, Nigeria, using selected physico-chemical parameters. *Turkish Journal of Fisheries and Aquatic Sciences*, *8*(2), 309–319.

Mustapha, M. K. (2009). Influence of watershed activities on the water quality and fish assemblages of a tropical African reservoir. *Turkish Journal of Fisheries and Aquatic Sciences*, *9*(1), 01–08. https://doi.org/10.15517/rbt.v57i3.5486

Nduka, J. K., & Orisakwe, O. E. (2011). Water-quality issues in the Niger Delta of Nigeria: A look at heavy metal levels and some physicochemical properties. *Environmental Science and Pollution Research*, *18*(2), 237–246. https://doi.org/10.1007/s11356-010-0366-3

Nganje, T. N., Edet, A., Cuthbert, S., Adamu, C. I., & Hursthouse, A. S. (2020). The concentration, distribution and health risk from potentially toxic elements in the soil - plant - water system developed on black shales in SE Nigeria. *Journal of African Earth Sciences*, *165*(February 2019), 103806. https://doi.org/10.1016/j.jafrearsci.2020.103806

Nganje, T. N., Hursthouse, A. S., Edet, A., Stirling, D., & Adamu, C. I. (2015). Assessment of the Health Risk, Aesthetic and Agricultural Quality of Rainwater, Surface Water and Groundwater in the Shale Bedrock Areas, Southeastern Nigeria. *Water Quality, Exposure and Health*, *7*(2), 153–178. https://doi.org/10.1007/s12403-014-0136-4

Nnorom, I. C., Ewuzie, U., & Eze, S. O. (2019). Multivariate statistical approach and water quality assessment of natural springs and other drinking water sources in Southeastern Nigeria. *Heliyon*, *5*(1), e01123. https://doi.org/10.1016/j.heliyon.2019.e01123

Ocheli, A., Otuya, O. B., & Umayah, S. O. (2020). Appraising the risk level of physicochemical and bacteriological twin contaminants of water resources in part of the western Niger Delta region. *Environmental Monitoring and Assessment*, *192*(5). https://doi.org/10.1007/s10661-020-08302-5

Odipe, O. E., Ogunleye, R. A., Sulaiman, M., Abubakar, S. S., & Olorunfemi, M. O. (2018). Integrated geophysical and hydro-chemical investigations of impact of the Ijemikin Waste Dump Site in Akure, Southwestern Nigeria, on groundwater quality. *Journal of Health and Pollution*, *8*(18). https://doi.org/10.5696/2156-9614-8.18.180604

Odukoya, A. M., Olobaniyi, S. B., Oluseyi, T. O., & Adeyeye, U. A. (2017). Health risk associated with some toxic elements in surface water of Ilesha gold mine sites, southwest Nigeria. *Environmental Nanotechnology, Monitoring and Management*, *8*(July), 290–296. https://doi.org/10.1016/j.enmm.2017.10.005

Odukoya, O. O., Onianwa, P. C., & Sanusi, O. I. (2010). Effect of highways and local activities on the quality of underground water in Ogun State, Nigeria : A case study of three districts in Ogun State, Nigeria. *Environmental Monitoring and Assessment*, *168*(1–4), 1–10. https://doi.org/10.1007/s10661-009-1086-3

Ogbeide, O., Chukwuka, A., Tongo, I., & Ezemonye, L. (2018). Relationship between geosorbent properties and field-based partition coefficients for pesticides in surface water and sediments of selected agrarian catchments: Implications for risk assessment. *Journal of Environmental Management*, *217*, 23–37. https://doi.org/10.1016/j.jenvman.2018.03.065

Ogbeide, O., Uhunamure, G., Okundaye, F., & Ejeomo, C. (2019). First report on probabilistic risk assessment of pesticide residues in a riverine ecosystem in South-South Nigeria. *Chemosphere*, *231*, 546–561. https://doi.org/10.1016/j.chemosphere.2019.05.105

Ogunbanwo, O. M., Kay, P., Boxall, A. B., Wilkinson, J., Sinclair, C. J., Shabi, R. A., Fasasi, A. E., Lewis, G. A., Amoda, O. A., & Brown, L. E. (2020). High Concentrations of Pharmaceuticals in a Nigeria River Catchment. *Environmental Toxicology and Chemistry*, 0–2. https://doi.org/10.1002/etc.4879

Ogunkunle, C. O., Mustapha, K., Oyedeji, S., & Fatoba, P. O. (2016). Assessment of metallic pollution status of surface water and aquatic macrophytes of earthen dams in Ilorin, north-central of Nigeria as indicators of environmental health. *Journal of King Saud University - Science*, *28*(4), 324–331. https://doi.org/10.1016/j.jksus.2015.11.005

Ogwueleka, T. C. (2014). Assessment of the water quality and identification of pollution sources of Kaduna River in Niger State (Nigeria) using exploratory data analysis. *Water and Environment Journal*, *28*(1), 31–37. https://doi.org/10.1111/wej.12004

Okafor, P. N., & Ogbonna, U. I. (2003). Nitrate and nitrite contamination of water sources and fruit juices marketed in South-Eastern Nigeria. *Journal of Food Composition and Analysis*, *16*(2), 213–218. https://doi.org/10.1016/S0889-1575(02)00167-9

Okorie, D. O., Eleazu, C. O., & Akabuogu, O. W. (2015). Quality evaluation of commercially sold table water samples in Michael Okpara University of Agriculture, Umudike, Nigeria and surrounding environments. *Toxicology Reports*, *2*, 904–907. https://doi.org/10.1016/j.toxrep.2015.05.016

Okunola, O. J., Oladipo, M. O. A., Aker, T., & Popoola, O. B. (2020). Risk assessment of drinkable water sources using gross alpha and beta radioactivity levels and heavy metals. *Heliyon*, *6*(8), e04668. https://doi.org/10.1016/j.heliyon.2020.e04668

Olajire, A. ., & Imeokparia, F. . (2001). Water Quality Assessment of Osun River. *Environmental Monitoring and Assessment*, *69*, 17–22. http://www.uclmail.net/users/dn.cash/EDTA1.pdf

Olaoye, O. A., & Onilude, A. A. (2009). Assessment of microbiological quality of sachet-packaged drinking water in Western Nigeria and its public health significance. *Public Health*, *123*(11), 729–734. https://doi.org/10.1016/j.puhe.2009.09.015

Olatunji, A. S., & Ajay, F. (2016). Potentially Toxic Contamination of Cultivated Wetlands in Lagos, Nigeria. *Journal of Health and Pollution*, *6*(10), 95–102. https://doi.org/10.5696/2156-9614-6.10.95

Ololade, I. A., Oladoja, N. A., Ololade, O. O., Oloye, F. F., Adeola, A. O., Alabi, A. B., Ademila, O., Adanigbo, P., & Owolabi, M. B. (2018). Geographical distribution of perfluorooctanesulfonate and perfluorooctanoate in selected rivers from Nigeria. *Journal of Environmental Chemical Engineering*, *6*(4), 4061–4069. https://doi.org/10.1016/j.jece.2018.06.020

Omaka, O. N., Aghamelu, O. P., Ike-Amadi, C. A., & Ofoezie, R. C. (2017). Assessment of the quality of groundwater from different parts of southeastern Nigeria for potable use. *Environmental Earth Sciences*, *76*(9), 1–24. https://doi.org/10.1007/s12665-017-6680-z

Omaka, O. N., Offor, I. F., Igwe, D. O., & Ugochukwu, E. (2016). Evaluation of groundwater quality of selected boreholes in Ohaozara and Ivo Council Areas of Ebonyi State, Nigeria. *Pakistan Journal of Scientific and Industrial Research Series A: Physical Sciences*, *59*(1).

Omo-Irabor, O. O., Olobaniyi, S. B., Oduyemi, K., & Akunna, J. (2008). Surface and groundwater water quality assessment using multivariate analytical methods: A case study of the Western Niger Delta, Nigeria. *Physics and Chemistry of the Earth*, *33*(8–13), 666–673. https://doi.org/10.1016/j.pce.2008.06.019

Omonona, O. V., Onwuka, O. S., & Okogbue, C. O. (2014). Characterization of groundwater quality in three settlement areas of Enugu metropolis, southeastern Nigeria, using multivariate analysis. *Environmental Monitoring and Assessment*, *186*(2), 651–664. https://doi.org/10.1007/s10661-013-3405-y

Omonona, Olufemi V., Amah, J. O., Olorunju, S. B., Waziri, S. H., Ekwe, A. C., Umar, D. N., & Olofinlade, S. W. (2019). Hydrochemical characteristics and quality assessment of groundwater from fractured Albian carbonaceous shale aquifers around Enyigba-Ameri, southeastern Nigeria. *Environmental Monitoring and Assessment*, *191*(3). https://doi.org/10.1007/s10661-019-7236-3

Onwuka, O. S., Umar, N. D., Omonona, O. V., & Idris, I. G. (2019). Heavy metals and rare earth elements distribution in the brine fields of awe, keana and giza, central benue trough, Nigeria. *Journal of African Earth Sciences*, *157*(October 2018), 103514. https://doi.org/10.1016/j.jafrearsci.2019.103514

Onyekwelu, I. L., & Aghamelu, O. P. (2019). Impact of organic contaminants from dumpsite leachates on natural water sources in the Enugu Metropolis, southeastern Nigeria. *Environmental Monitoring and Assessment*, *191*(9). https://doi.org/10.1007/s10661-019-7719-2

Opafola, O. T., Oladepo, K. T., Ajibade, F. O., & David, A. O. (2020). Potability assessment of packaged sachet water sold within a tertiary institution in southwestern Nigeria. *Journal of King Saud University - Science*, *32*(3), 1999–2004. https://doi.org/10.1016/j.jksus.2020.02.004

Orisakwe, O. E., Igwilo, I. O., Afonne, O. J., Maduabuchi, J. M. U., Obi, E., & Nduka, J. C. (2006). Heavy metal hazards of sachet water in Nigeria. *Archives of Environmental and Occupational Health*, *61*(5), 209–213. https://doi.org/10.3200/AEOH.61.5.209-213

Osinowo, O. O. (2016). Water quality assessment of the Asata River catchment area in Enugu Metropolis, Southeast Nigeria. *Journal of African Earth Sciences*, *121*(2016), 247–254. https://doi.org/10.1016/j.jafrearsci.2016.06.009

Owamah, H. I. (2020). A comprehensive assessment of groundwater quality for drinking purpose in a Nigerian rural Niger delta community. *Groundwater for Sustainable Development*, *10*(July 2019), 100286. https://doi.org/10.1016/j.gsd.2019.100286

Owoyemi, F. B., Oteze, G. E., & Omonona, O. V. (2019). Spatial patterns, geochemical evolution and quality of groundwater in Delta State, Niger Delta, Nigeria: implication for groundwater management. *Environmental Monitoring and Assessment*, *191*(10). https://doi.org/10.1007/s10661-019-7788-2

Paca, J. M., Santos, F. M., Pires, J. C. M., Leitão, A. A., & Boaventura, R. A. R. (2019). Quality assessment of water intended for human consumption from Kwanza, Dande and Bengo rivers (Angola). *Environmental Pollution*, *254*. https://doi.org/10.1016/j.envpol.2019.113037

Rim-Rukeh, A., Ikhifa, G. O., & Okokoyo, P. A. (2007). Physico-chemical characteristics of some waters used for drinking and domestic purposes in the Niger Delta, Nigeria. *Environmental Monitoring and Assessment*, *128*(1–3), 475–482. https://doi.org/10.1007/s10661-006-9340-4

Sawyerr, H. O., Adeolu, A. T., Afolabi, A. S., Salami, O. O., & Badmos, B. K. (2017). Impact of dumpsites on the quality of soil and groundwater in satellite towns of the Federal Capital Territory, Abuja, Nigeria. *Journal of Health and Pollution*, *7*(14), 15–22. https://doi.org/10.5696/2156-9614-7.14.15

Shu’aibu, H. K., Khandaker, M. U., Baballe, A., Tata, S., & Adamu, M. A. (2020). Determination of radon concentration in groundwater of Gadau, Bauchi State, Nigeria and estimation of effective dose. *Radiation Physics and Chemistry*, *February*, 108934. https://doi.org/10.1016/j.radphyschem.2020.108934

Singh, K. P., Malik, A., Mohan, D., & Sinha, S. (2004). Multivariate statistical techniques for the evaluation of spatial and temporal variations in water quality of Gomti River (India) - A case study. *Water Research*, *38*(18), 3980–3992. https://doi.org/10.1016/j.watres.2004.06.011

Sogbanmu, T. O., Osibona, A. O., & Otitoloju, A. A. (2019). Specific polycyclic aromatic hydrocarbons identified as ecological risk factors in the Lagos lagoon, Nigeria. *Environmental Pollution*, *255*, 113295. https://doi.org/10.1016/j.envpol.2019.113295

Sojobi, A. O. (2016). Evaluation of groundwater quality in a rural community in North Central of Nigeria. *Environmental Monitoring and Assessment*, *188*(3), 1–17. https://doi.org/10.1007/s10661-016-5149-y

Stephen, U. N., Celestine, O. O., & Solomon, O. O. (2017). Analysis of hydrogeochemical facies in groundwater of upper part of Cross River Basin, southeastern Nigeria. *Journal of African Earth Sciences*, *131*, 145–155. https://doi.org/10.1016/j.jafrearsci.2017.04.007

Talabi, A. O., Abdu-Raheem, Y. A., Afolagboye, L. O., Oguntuase, M. A., & Akinola, O. O. (2020). Hydrogeochemistry of shallow groundwater in Ado-Ekiti Area, Southwestern Nigeria. *Groundwater for Sustainable Development*, *11*(July 2019), 100386. https://doi.org/10.1016/j.gsd.2020.100386

Titilawo, Y., Adeniji, A., Adeniyi, M., & Okoh, A. (2018). Determination of levels of some metal contaminants in the freshwater environments of Osun State, Southwest Nigeria: A risk assessment approach to predict health threat. *Chemosphere*, *211*, 834–843. https://doi.org/10.1016/j.chemosphere.2018.07.203

Tongo, I., Ezemonye, L., & Akpeh, K. (2017). Levels, distribution and characterization of Polycyclic Aromatic Hydrocarbons (PAHs) in Ovia river, Southern Nigeria. *Journal of Environmental Chemical Engineering*, *5*(1), 504–512. https://doi.org/10.1016/j.jece.2016.12.035

Turajo, K. A., Abubakar, B. S. U. I., Dammo, M. N., & Sangodoyin, A. Y. (2019). Burial practice and its effect on groundwater pollution in Maiduguri, Nigeria. *Environmental Science and Pollution Research*, *26*(23), 23372–23385. https://doi.org/10.1007/s11356-019-05572-6

Ubuoh, E. A., Ezenwa, L. E., Ndukwu, M. C., & Emeka-Chris, C. C. (2019). Assessment of cation chemistry of groundwater near hospital wastes dumpsites in Umuahia Nigeria using multivariate and analytical index approach. *Environmental Technology and Innovation*, *15*, 100371. https://doi.org/10.1016/j.eti.2019.100371

Ugwoke, T. A. S., & Waziri, S. H. D. (2020). Variation of groundwater depth and cation concentrations with CBRs of residual soils: Case study of three lithologic terrains from North-central Nigeria. *Journal of African Earth Sciences*, *172*(July), 103965. https://doi.org/10.1016/j.jafrearsci.2020.103965

Ukah, B. U., Igwe, O., & Ameh, P. (2018). The impact of industrial wastewater on the physicochemical and microbiological characteristics of groundwater in Ajao- Estate Lagos, Nigeria. *Environmental Monitoring and Assessment*, *190*(4). https://doi.org/10.1007/s10661-018-6600-z

Usese, A., Chukwu, O. L., Rahman, M. M., Naidu, R., Islam, S., & Oyewo, E. O. (2017). Concentrations of arsenic in water and fish in a tropical open lagoon, Southwest-Nigeria: Health risk assessment. *Environmental Technology and Innovation*, *8*, 164–171. https://doi.org/10.1016/j.eti.2017.06.005

Uzoukwu, B. A., Ngoka, C., & Nneji, N. (2004). Monitoring of seasonal variation in the water quality of Ubu River in Ekwusigo and Nnewi local government areas of Anambra State, Nigeria. *Environmental Management*, *33*(6), 886–898. https://doi.org/10.1007/s00267-004-3058-2

Vincent-Akpu, I. F., Tyler, A. N., Wilson, C., & Mackinnon, G. (2015). Assessment of physico-chemical properties and metal contents of water and sediments of Bodo Creek, Niger Delta, Nigeria. *Toxicological and Environmental Chemistry*, *97*(2), 135–144. https://doi.org/10.1080/02772248.2015.1041526

Wang, J., Liu, G., Liu, H., & Lam, P. K. S. (2017). Multivariate statistical evaluation of dissolved trace elements and a water quality assessment in the middle reaches of Huaihe River, Anhui, China. *Science of the Total Environment*, *583*, 421–431. https://doi.org/10.1016/j.scitotenv.2017.01.088

Williams, A. B., & Benson, N. U. (2010). Interseasonal hydrological characteristics and variabilities in surface water of tropical estuarine ecosystems within Niger Delta, Nigeria. *Environmental Monitoring and Assessment*, *165*(1–4), 399–406. https://doi.org/10.1007/s10661-009-0955-0

Wu, Z., Lai, X., & Li, K. (2021). Water quality assessment of rivers in Lake Chaohu Basin (China) using water quality index. *Ecological Indicators*, *121*(September 2020), 107021. https://doi.org/10.1016/j.ecolind.2020.107021

Zacchaeus, O. O., Adeyemi, M. B., Azeem Adedeji, A., Adegoke, K. A., Anumah, A. O., Taiwo, A. M., & Ganiyu, S. A. (2020). Effects of industrialization on groundwater quality in Shagamu and Ota industrial areas of Ogun state, Nigeria. *Heliyon*, *6*(7), e04353. https://doi.org/10.1016/j.heliyon.2020.e04353

Zume, J. T. (2011). Assessing the potential risks of burial practices on groundwater quality in rural north-central Nigeria. *Journal of Water and Health*, *9*(3), 609–616. https://doi.org/10.2166/wh.2011.193
